# Crematoenones – a novel substance class exhibited by ants functions as appeasement signal

**DOI:** 10.1186/1742-9994-10-32

**Published:** 2013-06-06

**Authors:** Florian Menzel, Nico Blüthgen, Till Tolasch, Jürgen Conrad, Uwe Beifuß, Till Beuerle, Thomas Schmitt

**Affiliations:** 1 Department of Evolutionary Biology, University of Mainz, Institute of Zoology, Johannes-von-Müller-Weg 6, 55099, Mainz, Germany; 2 University of Darmstadt, Faculty of Biology, Institute of Zoology, Schnittspahnstrasse 3, 64287, Darmstadt, Germany; 3 University of Hohenheim, Institute of Zoology, Garbenstr. 30, 70593, Stuttgart, Germany; 4 University of Hohenheim, Institute of Chemistry, Garbenstr. 30, 70593, Stuttgart, Germany; 5 University of Technology Braunschweig, Institute of Pharmaceutical Biology, Mendelssohnstr. 1, 38106, Braunschweig, Germany; 6 Department of Evolutionary Biology and Animal Ecology, University of Freiburg, Faculty of Biology, Hauptstr.1, 79104, Freiburg, Germany; 7 Department of Animal Ecology and Tropical Biology, University of Würzburg, Biocenter, Am Hubland, 97074, Würzburg, Germany

**Keywords:** Appeasement substance, Cuticular hydrocarbons, Formicidae, Interspecific aggression, Nestmate recognition cues, Parabiotic association, Alkyloctahydronaphthalene

## Abstract

**Background:**

Parasitic, commensalistic, and mutualistic guests in social insect colonies often circumvent their hosts’ nestmate recognition system to be accepted. These tolerance strategies include chemical mimicry and chemical insignificance. While tolerance strategies have been studied intensively in social parasites, little is known about these mechanisms in non-parasitic interactions.

Here, we describe a strategy used in a parabiotic association, i.e. two mutualistic ant species that regularly share a common nest although they have overlapping food niches. One of them, *Crematogaster modiglianii*, produces an array of cuticular compounds which represent a substance class undescribed in nature so far. They occur in high abundances, which suggests an important function in the ant’s association with its partner *Camponotus rufifemur*.

**Results:**

We elucidated the structure of one of the main compounds from cuticular extracts using gas chromatography, mass spectrometry, chemical derivatizations and nuclear magnetic resonance spectroscopy (NMR). The compound consists of two fused six-membered rings with two alkyl groups, one of which carries a keto functionality. To our knowledge, this is the first report on the identification of this substance class in nature. We suggest naming the compound crematoenone.

In behavioural assays, crematoenones reduced interspecific aggression. *Camponotus* showed less aggression to allospecific cuticular hydrocarbons when combined with crematoenones. Thus, they function as appeasement substances. However, although the crematoenone composition was highly colony-specific, interspecific recognition was mediated by cuticular hydrocarbons, and not by crematoenones.

**Conclusions:**

Crematenones enable *Crematogaster* to evade *Camponotus* aggression, and thus reduce potential costs from competition with *Camponotus.* Hence, they seem to be a key factor in the parabiosis, and help *Crematogaster* to gain a net benefit from the association and thus maintain a mutualistic association over evolutionary time.

To our knowledge, putative appeasement substances have been reported only once so far, and never between non-parasitic species. Since most organisms associated with social insects need to overcome their nestmate recognition system, we hypothesize that appeasement substances might play an important role in the evolution and maintenance of other mutualistic associations as well, by allowing organisms to reduce costs from antagonistic behaviour of other species.

## Introduction

The integrity of a social insect colony requires that the colony members only allow nestmates access to the nest and fend off other individuals or species. This is crucial to insure that the insect colony is not invaded by parasites. The discrimination between nestmate and non-nestmate, and hence, the choice between tolerance and aggression between two encountering individuals, usually depends on the match or mismatch of their cuticular hydrocarbon profiles [1,2]. However, certain intruding species circumvent this system to avoid being attacked. For example, many guests of social insects (myrmecophiles) or social parasites manipulate their host’s nestmate recognition system by chemical mimicry (mimicking the recognition cues of the host) or chemical insignificance (reducing the perceptibility or quantity of their own recognition cues, [3]). There is one report that a social parasite putatively achieves (or reinforces) host acceptance through appeasement allomones [4]. Alternatively, ‘propaganda allomones’ can elicit panic among the hosts and thereby prevent the parasite from being attacked [3,5].

**Figure 1:**
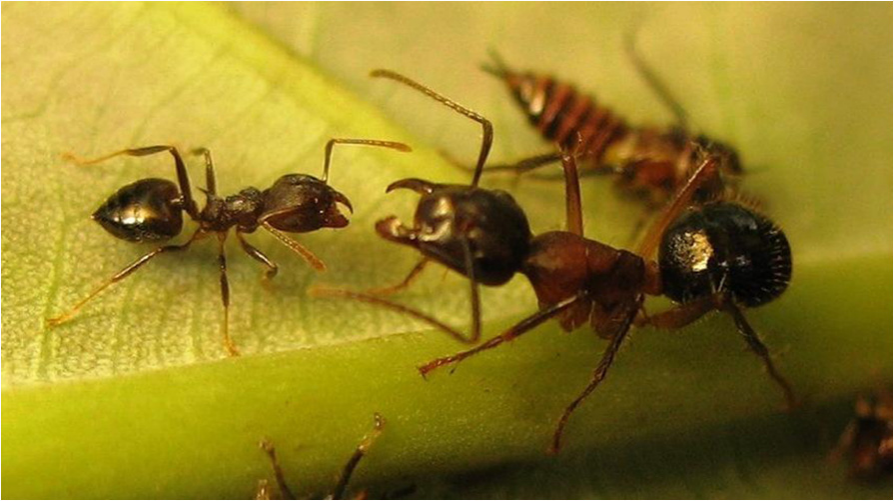
**The two parabiotic ant species, *Crematogaster modiglianii *(left) and *Camponotus rufifemur *(right).**

A peculiar case of two social insects living together is the parabiosis, where a colony consists of two ant species which inhabit the same nest and forage peacefully together, but keep their brood separately [6]. This type of association is rare and has been described only for a few pairs of species in the world. Previous studies showed that the paleotropical parabiosis between *Camponotus rufifemur* and *Crematogaster modiglianii* (Figure 1) and the neotropical parabiosis between *Camponotus femoratus* and *Crematogaster levior* are mutualistic [7,8]. This makes parabioses an interesting model system for studies on interspecific recognition: in contrast to host–parasite associations, both partners should have an interest in maintaining the association. In the paleotropical parabiosis, both *Crematogaster* and *Camponotus* are indeed remarkably tolerant towards each other and show little aggression even towards non-nestmate members of the partner species [9]. This raises the question of why the ability to discriminate between different colonies of the partner species (and in part, of their own species) is so low.

Previous studies on nestmate recognition in these species revealed that *Ca. rufifemur* has an unusual cuticular hydrocarbon profile, which might lead to reduced nestmate discrimination ability by its parabiotic partner *Cr. modiglianii*. While different from *Cr. modiglianii*, the profile of *Ca. rufifemur* (which occurs in a ‘red’ and a ‘black’ variety) has significantly higher chain lengths than other, congeneric ant species [10,11]. Hydrocarbons with extremely long carbon backbones are probably harder to perceive, and may thus provide fewer recognition cues than shorter ones [12]. Indeed, intraspecific nestmate recognition in *Ca. rufifemur* is very low, and all investigated parabiotic *Camponotus* species show these specific alterations in their cuticular profiles [10,11]. A second peculiarity of both parabiotic ants, however, is that the cuticle of *Cr. modiglianii* contains a set of polar compounds, which are highly abundant in the species *Cr. modiglianii* and are, in smaller amounts, transferred to *Ca. rufifemur*[10]. These polar compounds were tentatively identified as steroids in our previous study; however, the detailed characterization presented here indicates a different molecular structure. The relative composition of these compounds is highly variable among different parabiotic nests, but shows similarities between the two ant species within a nest [10,13]. This makes them suitable as potential recognition cues, and could in theory allow discrimination between intra- and allocolonial individuals of their own and the partner species based on the same cuticular substances.

Cuticular substances that are neither hydrocarbons nor hydrocarbon derivatives are highly unusual in arthropods (although recently, triacylglycerides were found from insect cuticles [14,15]). Since nestmate recognition is usually mediated by cuticular substances, the two described peculiarities raise the question how interspecific tolerance is achieved among seemingly equal partners, and what role hydrocarbons and novel compounds play in the interspecific recognition process. The hydrocarbons, which usually function as recognition cues, may be harder to perceive than those in non-parabiotic species, and thus less suitable as recognition cues [11]. Recognition might be mediated by the novel substances, which seem suitable as recognition cues. As an alternative function, they may have an appeasing effect, and be responsible for the low *Camponotus* aggression towards the much smaller *Crematogaster*[9].

In the present study, we characterize the molecular structure of the novel compounds and show that they represent a novel substance class which has not been found previously in nature, and for which we suggest the name ‘crematoenones’. We subsequently identify the role of hydrocarbons vs. crematoenones in the nestmate recognition process. The results of our behavioural assays indicate that the hydrocarbons function as recognition cues by which the partner species is recognized. The novel compounds, in contrast, are not used as recognition cues. However, they act as appeasement substances and reduce aggressiveness of *Ca. rufifemur* towards hydrocarbons of non-nestmates, or even other, non-parabiotic ant species. Thus, the two mechanisms – long-chain hydrocarbons and appeasement substances seem to act jointly to maintain tolerance between parabiotic species.

## Results

### Structure of the novel compounds

Overall, 24 novel non-hydrocarbon compounds were found on the cuticle of *Cr. modiglianii* from seven colonies (Additional file 1: Table S1; Additional file 2: Figure S1). Their overall abundance exceeded the hydrocarbon abundance by the factor 5.70 ± 1.39 SE (ratios of total ion counts; n = 11 colonies). All novel compounds possessed eight similar diagnostic ions, which at most differed by 2 mass units (ions 41/43, 55, 67/69/71, 79, 91, 121, 135, and 231/233). Three further ions were present in the majority of the 24 compounds (ions 201/203, 259, and 300/302; Additional file 2: Figure S6). An initial comparison of their electron ionization mass spectra with mass spectra from commercial libraries had shown high accordance with a basic steroid structure [10]. In our previous study [10], we had therefore misinterpreted the mass spectra as indicative of a basic steroid structure. However, the results of HRMS as well as mass spectra of the hydrogenated compounds do not support this initial tentative interpretation (Additional file 3: Table S3).

**Figure 2:**
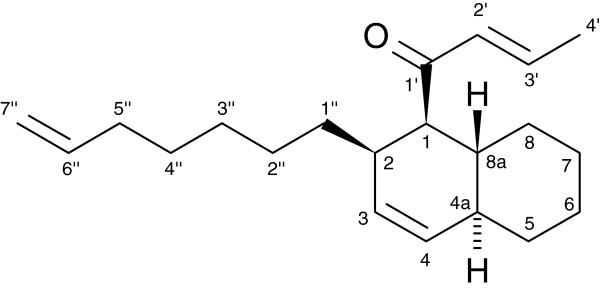
Chemical structure of crematoenone (compound 10).

The molecular structure of compound 10 (Additional file 1: Table S1; Additional file 2: Figure S2; Additional file 3) was further analysed. This compound occurred in all seven investigated colonies and, in five colonies, represented the single most abundant compound. Based on an extract of several tens of thousands of ants, we obtained ^1^H and ^13^C NMR data and ROESY and HMBC correlations (including relative configurations) (Additional file 3: Table S4). The structure elucidation (see Additional file 3) revealed an octahydronaphthalene subunit with a 2-butenoyl substituent as well as an alkenyl moiety with a terminal double bond (Figure 2). Thus, the IUPAC name of compound 10 is (*E*)-1-((1R*,2R*,4aS*,8aR*)-2-(hept-6-enyl)-1,2,4a,5,6,7,8,8a-octahydro-naphthalene-1-yl)-but-2-en-1-one, and we suggest the trivial name ‘crematoenone’.

The remaining 23 substances occurred on average in far lower abundances, which did not allow NMR analysis. However, based on their similar mass spectra, we tentatively assume that they are structurally related (Additional file 2: Figure S6). Beside the name ‘crematoenone’ for compound 10 we therefore suggest to use the plural form ‘crematoenones’ for the whole substance class, reflecting the single double bond in the octahydronaphtalene subunit and the keto moiety.

For two further compounds (no. 6 and 18 in Additional file 1: Table S1; Additional file 2: Figure S2; Additional file 3), we obtained enough substance to conduct HRMS analysis, and based on the reconstruction and extrapolation of EI fragmentation mechanisms, we can propose sound chemical structures for these compounds (Additional file 2: Figures S2, S3, S4 and S5). Compound 6 was tentatively identified as 2′,3′-dihydrocrematoenone. In compound 18, the double bond of the alkenyl moiety was probably replaced by an O-acetyl functionality, i.e. compound 18 would be an O-acetyldihydrocrematoenone.

### Behavioural assays: ***Camponotus*** towards ***Crematogaster***

The behavioural assays were to determine the role of hydrocarbons vs. crematoenones in nestmate recognition. We confronted a *Camponotus rufifemur* colony (black variety; colony B1) with dead *Crematogaster modiglianii* workers from its partner colony (intracolonial) and from a non-nestmate *Cr. modiglianii* colony (allocolonial), and measured whether the *Ca. rufifemur* aggression differed between intracolonial and allocolonial *Cr. modiglianii*. In three subsequent treatments, we similarly determined whether *Ca. rufifemur* distinguished their partner from the non-nestmate colony, but the *Ca. rufifemur* ants were only confronted with cues, presented on odourless ‘dummies’ (dead, solvent-washed ants). These cues were (1) whole cuticular extracts, (2) cuticular hydrocarbons only, and (3) crematoenones only, each time from intra- and allocolonial *Cr. modiglianii.* The cuticular hydrocarbons of different *Cr. modiglianii* colonies show only quantitative differences; however, those colonies living together with the red *Ca. rufifemur* variety possess two hydrocarbons that are absent from those living with the black variety [13].

**Figure 3:**
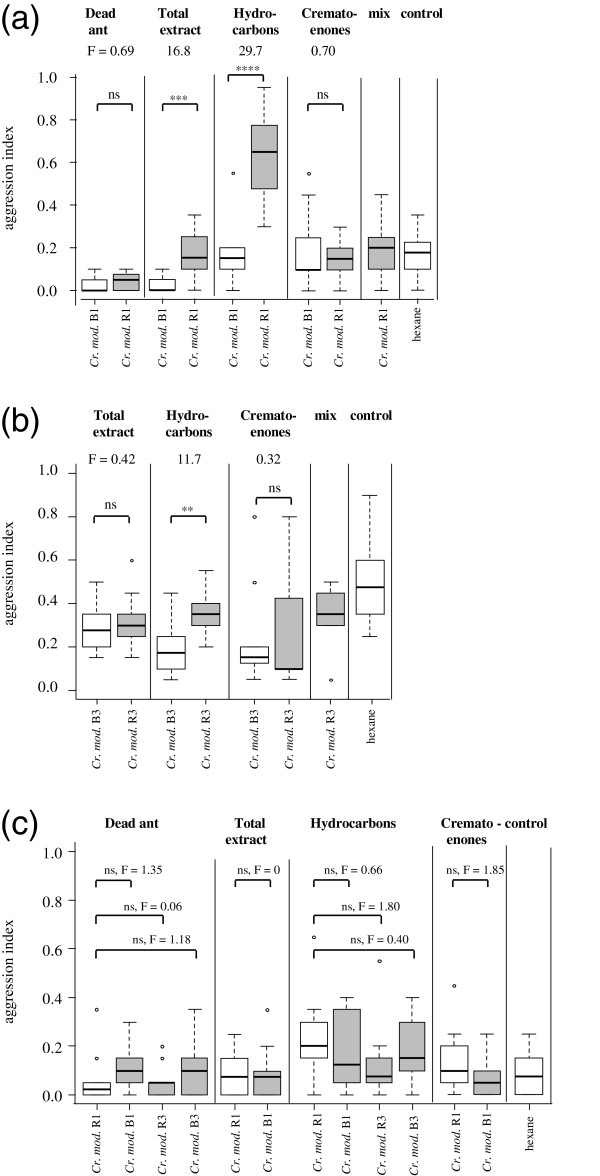
**Aggression of two *Camponotus rufifemur *colonies ((a) colony B1, (b) colony B3), (c) colony R1) towards intracolonial and allocolonial *Crematogaster modiglianii*.** Dead ants, total cuticular extracts, cuticular hydrocarbons and cuticular crematoenones were tested. Each plot represents 10 replicates. ‘mix’: Hydrocarbons (non-polar fraction) and crematoenones (polar fraction) mixed. ‘control’: Dummies treated with pure hexane. Intracolonial treatments and hexane controls are represented by empty plots, allocolonial treatments by grey plots. Effect sizes (F values) are given above each pairwise comparison. Asterisks denote significant differences according to pairwise GLM comparisons (all df = 1). ***p < 0.0001, **p < 0.01, *p < 0.05, ‘n.s.’ p > 0.1.

*Camponotus rufifemur* did not differentiate between dead intracolonial and dead allocolonial *Cr. modiglianii* workers. However, they discriminated between their extracts and especially between their hydrocarbon fractions. Cuticular extracts of allocolonial *Cr. modiglianii* elicited significantly more aggression than intracolonial ones. The hydrocarbon fractions of allocolonial *Cr. modiglianii* (R1, living with the red *Ca. rufifemur* variety) triggered very high aggression, while hydrocarbon fractions of intracolonial *Cr. modiglianii* were treated amicably (Figure 3a). This differential aggression was highly significant. In contrast, the behaviour towards the crematoenone fractions was mainly peaceful for both intracolonial and allocolonial cues. Similarly, a re-mixture of hydrocarbon and crematoenone fractions of allocolonial *Cr. modiglianii* received little aggression, which corresponds to the weaker differentiation between the two total extracts compared to the two hydrocarbon fractions.

A black *Ca. rufifemur* worker colony from a different parabiosis (B3) showed comparable behaviour, significantly differentiating between hydrocarbon fractions of intra- and allocolonial *Cr. modiglianii* but not between their total extracts or their crematoenone fractions (Figure 3b). In contrast to the two black *Ca. rufifemur* colonies (B1 and B3), however, a red *Ca. rufifemur* colony (R1) never showed higher aggression towards allocolonial *Cr. modiglianii* treatments. The red *Ca. rufifemur* workers were confronted with dead workers, total extracts, hydrocarbons, and crematoenones of one non-nestmate *Cr. modiglianii* colony, and with dead workers and hydrocarbons of two further non-nestmate *Cr. modiglianii* colonies. In no case did the red *Ca. rufifemur* workers show significant differentiation from the intracolonial *Cr. modiglianii* (Figure 3c).

**Figure 4:**
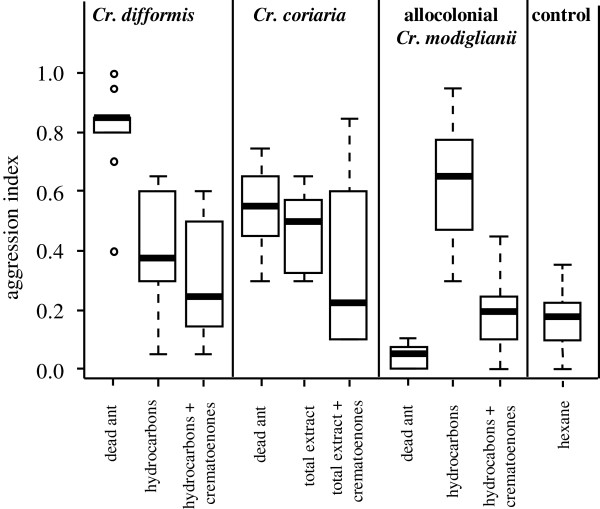
**Effect of crematoenone addition on aggression of *Camponotus rufifemur *(colony B1).** Dead ants, cuticular hydrocarbons, and cuticular hydrocarbons mixed with allocolonial *Cr. modiglianii* crematoenones were tested. For *Cr. coriaria*, cuticular extracts only contained hydrocarbons but no other substances. Each plot represents 10 replicates. The third and the forth panel contains data also shown in Figure 3a.

The addition of allocolonial *Cr. modiglianii* crematoenones to different extracts significantly reduced aggressiveness of *Ca. rufifemur* workers. They strongly attacked dead bodies of *Crematogaster coriaria* and *Cr. difformis*, as well as their cuticular hydrocarbons and those of allocolonial *Cr. modiglianii*. However, *Ca. rufifemur* was significantly less aggressive to each of these treatments after addition of allocolonial *Cr. modiglianii* crematoenones (Figure 4, Additional file 1: Table S2). The effect was significantly higher for *Cr. modiglianii* extracts than for the other two species (significant interaction ‘*Crematogaster* species’: ‘crematoenone addition’, Additional file 1: Table S2), but still show a trend when only *Cr. coriaria* and *Cr. difformis* were considered (GLM with *Cr. coriaria* and *Cr. difformis* only: F_1,39_ = 2.9, *P* = 0.09).

### Behavioural assays: *Crematogaster* towards *Camponotus*

**Figure 5:**
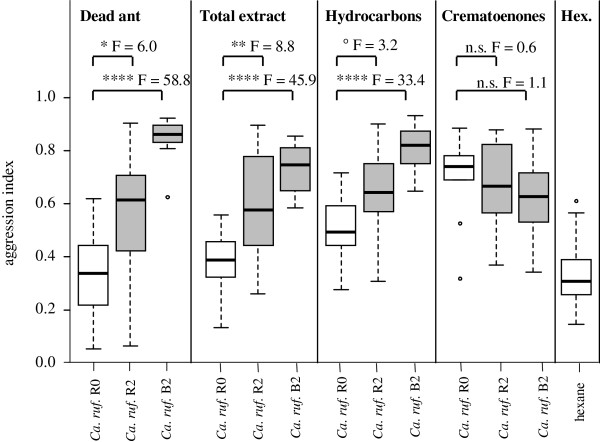
**Aggression of *Crematogaster modiglianii *(colony R0) against intracolonial *Ca. rufifemur *R0 (white boxes), allocolonial red *Ca. rufifemur *R2 and allocolonial black *Ca. rufifemur *B2 (grey boxes each).** Dead ants, total cuticular extracts, cuticular hydrocarbons and cuticular crematoenones were tested. Each plot represents 10 replicates. ‘control’: Dummies treated with pure hexane. Intracolonial treatments and hexane controls are represented by empty plots, allocolonial treatments by grey plots. Effect sizes (F values) are given above each pairwise comparison. Asterisks denote significant differences according to pairwise GLM comparisons (all df = 1). ***p < 0.0001, **p < 0.01, *p < 0.05, ‘n.s.’ p > 0.1.

In a similar test series, we confronted a *Crematogaster modiglianii* (colony R0, living with a red *Ca. rufifemur*) with intra- and allocolonial *Ca. rufifemur* treatments. The workers attacked allocolonial dead workers of *Ca. rufifemur* significantly more than intracolonial ones. A similar, significant differentiation was found for whole cuticular extracts, and also for their hydrocarbon fractions. Here, aggression against black *Ca. rufifemur* treatments was much higher than against those of red *Ca. rufifemur* (Figure 5). In contrast, the crematoenone fractions did not trigger any significant differentiation between intra- and allocolonial treatments. In the test series for both *Cr. modiglianii* and *Ca. rufifemur*, all intracolonial treatments elicited aggression levels comparable to or lower than hexane controls (Figures 3, 4 and 5).

## Discussion

### A novel substance class produced by *Crematogaster*

The cuticle of insects is usually covered with non-polar hydrocarbons [16]. Only few insects possess oxygenated hydrocarbon derivatives, and most of these are species other than ants [17]. Cuticular substances that are neither hydrocarbons nor hydrocarbon derivatives are highly unusual in insects (but see [14,15]). The parabiotic ant *Crematogaster modiglianii* possesses a whole set of interrelated compounds on its cuticle. In our previous study, we could show that these substances are likely to be produced in the Dufour’s gland and from there spread onto the whole cuticle [10].

We characterized the molecular structure of the novel compounds using EI-MS, CI-MS, HRMS and NMR. Searches in the CAS database based on similarity or substructure motifs of compound 10 revealed that this class of chemicals has not been described for insects nor have they, to the best of our knowledge, been described in nature at all. Based on compound 10, we suggest to name this substance class ‘crematoenones’, reflecting the source genus *Crematogaster*, the double bond in the octahydronaphthalene subunit, and the keto function.

### Possible biosynthesis of the crematoenones

The genus *Crematogaster* is known for its ability to produce a wide array of structurally diverse chemicals. [10,18-23]. Compared to other ant species, the *Crematogaster* species studied so far possess a peculiar system of venom production: precursors from Dufour’s gland are derivatized by enzymes from the poison gland [21,24]. It may be due to this mechanism that *Crematogaster* species produce such chemically diverse venoms. While several of the previously described *Crematogaster* venoms are derived from either fatty acid or terpenoid metabolism, other ant venoms studied so far are often alkaloids and are probably derived from the amino acid, polyacetate, or fatty acid metabolism [25].

We propose that the biosynthesis of crematoenones can be linked to a polyketide-type biosynthesis pathway. A proposed polyketide of 11 acetate units, re-arranged by two intramolecular aldol condensations (finally forming the octahydronaphthalene ring system) followed by several modifications including decarboxylation, eliminations of water (resulting in double bonds) and multiple reductions could explain the formation of the C21-backbone of crematoenones. Biosynthesis based on a C21-hydrocarbon is an alternative pathway, but it seems less likely. Notably, the crematoenones seem to be synthesized *de novo*, since their composition remained constant in colonies that were kept in the laboratory on an artificial diet of dead insects, honey, and Bhatkar diet for up to 15 months [10]. This contrasts with e.g. herbivorous beetles, which take up toxins from their host plants with few if any modifications [25], although a fungal or microbial origin of crematoenones cannot be ruled out.

### Interspecific nestmate recognition is mediated by hydrocarbons

Among the seven colonies that were chemically characterized, we found a total of 24 different novel compounds. Although *Cr. modiglianii* exhibits colony-specific crematoenone profiles, neither of the two species differentiated between intra- and allocolonial crematoenones, but clearly discriminated intracolonial from allocolonial hydrocarbons of the respective partner species. Our experiments hence show that the recognition of the partner species (though not necessarily the partner colony) is mediated by cuticular hydrocarbons like it is known from other ant species [26], and not the crematoenones.

Interestingly, the relative composition varied strongly among *Cr. modiglianii* colonies (Additional file 1: Table S1, [13]), and this variance remained constant in wild and captive colonies [10]. The crematoenone profile might hence be highly genetically determined. However, since nestmate recognition is mediated through cuticular hydrocarbons (which are highly similar among the *Cr. modiglianii* colonies studied, [10,13]), they may also serve as sex pheromones like in many solitary insects [27,28]. Thus, differing crematoenone profiles should not lead to reproductive isolation. In preliminary analyses, we did not find genetic differentiation between *Cr. modiglianii* colonies according to crematoenone profile (FM unpublished data), providing no evidence for cryptic species in *Cr. modiglianii.*

The black *Ca. rufifemur* often, but not always differentiated between nestmate and non-nestmate *Cr. modiglianii* (based on dead workers, or whole cuticular extracts). In an earlier study [10], this was true even for living *Cr. modiglianii*. *Ca. rufifemur* sometimes failed to discriminate nestmate and non-nestmate, and we hypothesize that this was due to inter-colony variation in chemical distances among *Cr. modiglianii*, and to the long-chain hydrocarbons in *Cr. modiglianii*[10], which may hamper inter-colony discrimination. In contrast, the red *Ca. rufifemur* variety did not differentiate between nestmate and non-nestmate *Cr. modiglianii* treatments (see also [10]). Note that the *Cr. modiglianii* colonies living with a red *Ca. rufifemur* and those living with black *Ca. rufifemur* possessed *qualitatively* different hydrocarbon profiles: the former ones possessed the two main cuticular compounds of the red *Ca. rufifemur* (27-MeC_39_-14-ene and 27-MeC_39_-16-ene), which are absent in the latter ones [13]. Thus, it seems plausible that the black *Ca. rufifemur* can recognize the presence of these two compounds in non-nestmate *Cr. modiglianii*, whereas the red *Ca. rufifemur* cannot sense their absence, and hence fails to discriminate nestmate from non-nestmate *Cr. modiglianii*.

### Crematoenones reduce interspecific aggression

Though not as recognition cues, crematoenones play an important role in interspecific interactions by reducing *Ca. rufifemur* aggressiveness. While black *Ca. rufifemur* showed low aggression towards allocolonial *Cr. modiglianii*[9,10] or its surface extracts, their hydrocarbons alone elicited fierce attacks. When we re-added the previously removed crematoenones to the allocolonial hydrocarbon fractions, the resulting aggression of *Ca. rufifemur* was reduced to an intracolonial level. A similar, albeit weaker effect was found with extracts of two other *Crematogaster* species. While *Ca. rufifemur* usually attacked dummies with these extracts, their aggression levels were lower after crematoenone addition.

Thus, the crematoenones seem to function as appeasement substances. It is difficult to determine whether crematoenones reduce aggression at a neuronal level (i.e. tolerance despite recognition as non-nestmate) or whether they mask the recognition cues, i.e. hamper recognition itself at the receptor level. The former hypothesis implies that they act as behaviour modifiers, in analogy to e.g. honeybee queen pheromones in intraspecific signalling [29,30]. However, a definite distinction between these two possibilities will only be possible based on experiments that involve other behavioural responses than aggression, e.g. by testing whether crematoenone addition to intra- and allocolonial brood affects brood care, or by trying to condition ants on certain crematoenones [31].

Both *Camponotus rufifemur* and *Crematogaster modiglianii* possess cuticular hydrocarbons of significantly higher chain length than observed in non-parabiotic species. This shift in chain length is peculiar to closely associated ant species and seems to promote interspecific tolerance. Apparently, the recognition of quantitative (but not qualitative) differences (e.g. between conspecific colonies) is hampered in long-chain hydrocarbon profiles (probably due to their low volatility), at least for *Camponotus*[10,11]. The crematoenones seem to reduce aggression by impairing recognition of qualitative differences as well, thereby complementing the chemical tolerance mechanisms between the parabiotic partners.

Interestingly, the red *Ca. rufifemur* variety did not show any aggression even towards allocolonial *Crematogaster* workers. Hence, appeasement allomones would not be necessary here. However, the red and the black *Ca. rufifemur* varieties are sympatric and occur at similar abundances. Parabioses probably originate from *Cr. modiglianii* nests being colonised by *Ca. rufifemur*[7]*.* Since *Cr. modiglianii* is parabiotic with both *Ca. rufifemur* varieties, it may not be able to influence whether it is colonised by a black or a red *Ca. rufifemur*. Hence, it should produce appeasement allomones to allow a beneficial colonisation by both varieties, even if they are only necessary for a parabiosis with the black variety.

### Ecological and evolutionary implications

To date, the use of appeasement substances has been reported only for one other ant-ant association. The slave-making ant *Polyergus rufescens* uses decyl butyrate from its Dufour’s gland to calm its host’s aggression during host-colony usurpation [4,32]. However, this appeasement function is controversial since decyl butyrate acted as a repellent in a further study, thus suggesting repellence rather than appeasement [33]. In contrast, crematoenones appease rather than repel since *Ca. rufifemur* does not avoid contact with *Cr. modiglianii* workers (e.g. when foraging) and even approaches them for trophallaxis [7]. This would not be the case if they were repellent [33]. Although certain other social parasites use ‘propaganda’ substances to elicit panic among their hosts [3,5], we are not aware of any other case of appeasement substances among ants. Recently, however, sesquiterpenes that were not synthesized *de novo,* but acquired from the environment, were reported to have a calming effect on other species in communally nesting stingless bees [34]. Since the parabiosis is beneficial for both ant species, the appeasement benefits both on the long term.

*Camponotus rufifemur* and *Cr. modiglianii* share a nest, and each species tolerates the other completely, including the brood [7]. *Camponotus rufifemur* is a species of relatively large ants that easily drives away other ants from its nest [7] and from food resources (FM pers. obs.). However, it tolerates the much smaller *Crematogaster modiglianii*[7] but attacks other *Crematogaster* species [9]. As the present study reveals, this tolerance is likely caused by crematoenones (although a definite statement would only be possible with synthetic crematoenones). Interestingly, *Ca. rufifemur* does not defend itself if it is attacked by allocolonial *Cr. modiglianii*[9], which additionally argues for their appeasing function. The evolution of these appeasement substances presumably enabled *Cr. modiglianii* to share a nest with *Ca. rufifemur* instead of being displaced. This is beneficial for *Cr. modiglianii* since it can take advantage of additional nest space provided by *Ca. rufifemur* through wood excavations and carton constructions [7].

*Camponotus rufifemur* uses *Cr. modiglianii* trails to find food sources [35], and possibly also to find a *Cr. modiglianii* nest [7]. The two species forage together without overt aggression although they have overlapping food niches and thus are potential competitors for food [36]. Thus, the crematoenones may also benefit *Cr. modiglianii* in that they prevent it from being displaced from food. Otherwise, *Ca. rufifemur* would essentially parasitize on *Cr. modiglianii,* following its pheromone trails to food sources and then displacing *Cr. modiglianii* foragers. These hypotheses match other cases of mutualisms that evolved from parasitic associations where the host acquired adaptations to cope with the parasite [37,38].

Interestingly, in all reports on parabiotic associations, one of the two parties is a *Crematogaster* species [39]. The partner species, i.e. members of *Camponotus, Odontomachus, Pachycondyla* or *Dolichoderus*[39], are essentially all larger, and therefore probably more competitive, than *Crematogaster*. In the neotropics, a similar parabiosis exists between *Camponotus femoratus* and *Crematogaster levior*[8]. This association is also characterized by high interspecific tolerance between the two species, and low inter-colony discrimination (FM and TS, unpublished data). Similar to *Cr. modiglianii*, *Cr. levior* produces non-hydrocarbon cuticular substances (FM and TS, unpublished data). However, their structure and function are not elucidated yet. It seems possible that, as in the case studied here, the *Crematogaster*-specific ability to synthesize additional cuticular compounds with appeasement or similar functions was crucial for the evolution and maintenance of these parabioses.

## Conclusions

*Crematogaster modiglianii* produces a substance class (‘crematoenones’) that was unknown from nature until now. In behavioural assays, crematoenones reduced aggression of *Camponotus rufifemur*. Since *Cr. modiglianii* and *Ca. rufifemur* live and forage together despite being food competitors [7,35,36], it seems likely that crematoenones help *Cr. modiglianii* to evade *Ca. rufifemur* aggression, including displacement from the nest or from food resources. Interestingly, appeasement has been reported only once so far in a social parasite [4,32], and a later study showed repellence rather than appeasement in this system [33]. From an evolutionary point of view, an appeasement signal should only be stable if its receiver has a long-term benefit from being appeased. Otherwise, it is likely to evolve counter-adaptations against this signal. Thus, appeasement substances are evolutionarily stable only if neither of the species has a net cost from the association, i.e. in commensalisms or mutualisms. This is similar to the environmentally acquired substances with a calming effect in stingless bees [34]. Thus, it seems unlikely that true appeasement substances occur in host-parasite interactions. However, we hypothesize that they might play an important role in the initiation and maintenance of parabioses and other non-parasitic interactions.

## Materials and methods

### Study site and ants

Our experiments were conducted at Danum Valley Conservation Area from September to December 2007. Danum Valley is located at approximately 100 m a.s.l. in Sabah (Malaysian Borneo) and represents one of the major remaining patches of Sabah’s primary lowland rainforest. It has a typical equatorial rainforest climate with a mean annual temperature of 27°C and a yearly rainfall of 2700 mm. We studied parabioses of *Crematogaster modiglianii* and *Camponotus rufifemur* (Figure 1), which nest in hollow tree trunks [7]. *Camponotus rufifemur* occurs in two chemically and genetically distinct varieties, which probably represent different species (‘red’ and ‘black’ variety; [10,40]). In contrast, the profiles of the associated *Cr. modiglianii* had almost no compounds in common with *Ca. rufifemur* and did not show differentiation into chemical varieties, with two exceptions: 27-MeC_39_-14-ene and 27-MeC_39_-16-ene, the main surface components of the red *Ca. rufifemur*, occurred in those *Cr. modiglianii* colonies that lived in parabiosis with the red *Ca. rufifemur* but was absent from others [10,13].

Behavioural experiments were conducted with two nests (B1, R0) and one worker colony (B3) that were brought to the laboratory at Danum Valley, where both species were kept together inside a section of their original nest trunk in a Fluon™-covered plastic box for ca. two months (nests) or few days (worker colony), respectively. For the experiments, further ants were collected from four additional nests (B1, R1, R2, R3). Here, we use the term ‘nest’ for whole nests inside a living tree trunk, while ‘worker colony’ refers to groups of workers that were caught at a parabiotic nest. According to the *Ca. rufifemur* variety, the parabiotic colonies will be labelled ‘B’ or ‘R’ plus a digit in the following.

### Chemical analyses

We analysed the novel compounds from *Cr. modiglianii* cuticular extracts using electron ionization mass spectrometry (EI-MS), chemical ionization mass spectrometry (CI-MS), high resolution mass spectrometry (HRMS), and nuclear magnetic resonance (NMR). In addition, several derivatizations were performed and subsequently analysed with GC-MS (EI-MS). Firstly, we characterized different substances and their relative quantities from seven *Cr. modiglianii* colonies by their electron ionization mass spectra. Extracts were obtained from 20–90 individuals per extract, and we analysed 1–8 (Ø 3.5) extracts per colony. We performed capillary gas chromatography–mass spectrometry (GC-MS) with a Hewlett Packard 6890 series gas chromatograph coupled to a HP 5973 Mass Selective Detector. The GC was equipped with a J&W Scientific DB-5 fused silica capillary column (30 m × 0.25 mm ID; df = 0.25 μm). The temperature of the GC was kept at 60°C for 2 min, then increased by 60°C/min up to 200°C and subsequently by 4°C/min to 320°C, where it remained constant for 10 min. The transfer line had a temperature of 325°C. Helium was used as carrier gas with a constant flow of 1.0 ml/min. A split/splitless injector was installed at 250°C in the splitless mode for 30 s. The EI-MS were recorded with a ionization voltage of 70 eV and a source temperature of 230°C. The software MSD ChemStation (Version A.03.00) for Windows was used for data acquisition. Linear retention indices were calculated using Kovats’ method by linear interpolation from a series of *n-*alkanes.

Further chemical characterization was done for three main compounds among the novel compounds. Since most other compounds were represented by < 1% of all novel substances (Additional file 1: Table S1), we could not obtain enough extracts to unambiguously identify all remaining compounds. We obtained CI-MS with a Hewlett Packard 5890A gas chromatograph equipped with a 2 m fused silica guard column (deactivated, I.D. 0.32 mm) and a 30 m × 0.32 mm analytical column (ZB1 and ZB5, Phenomenex). The capillary column was directly coupled to a triple quadrupole mass spectrometer (TSQ 700, Finnigan). Injector and transfer line were kept at 280°C. Temperature was kept at 70°C for 3 min and then increased at 10°C/min up to 310°C, where it remained constant for five min. The CI mass spectra were recorded in the positive mode using ammonia as a reagent gas.

For HRMS, an Agilent 6890 gas chromatograph was equipped with a 30 m analytical column (Phenomenex ZB5-MS, 30 m × 0.25 mm ID, t_f_ = 0.25 μm). A split injection port at 250°C was used for sample introduction with a split ratio of 3:1. The temperature program was the same as for CI-MS. The helium carrier gas was set to 1.0 ml/min flow rate (constant flow mode). The transfer line was kept at 270°C. HRMS were acquired using a JMS-T100GC (GCAccuTOF, JEOL, Japan) time of flight MS (TOF-MS) in EI mode at 70 eV and JEOL MassCenter™ workstation software. Source and transfer line temperature were 200°C and 270°C, respectively, and detector voltage was set at 2100 V. The acquisition range was m/z 41 to 600 with a spectrum recoding interval of 0.4 s. The system was tuned with PFK to achieve a resolution of 5,000 (FWHM) at m/z 292.9824, and the mass accuracy across all suitable ions was better than 4 mmu.

NMR analyses were conducted using hexane extracts of several tens of thousands of ants. The extracts were purified using conditioned SiOH columns (CHROMABOND, 100 mg, Macherey-Nagel, Düren, Germany) with distilled hexane and chloroform as respective eluents. The novel compounds were eluted with chloroform, which was then evaporated and the fraction reconstituted in hexane. After concentration to 50 μl, the fraction was chromatographed over 2.0 g silica gel (Kieselgel 60, 0.04-0.063 mm, Carl Roth GmbH, Karlsruhe, Germany) in 12 fractions of 4 ml each, using the following solvents (pentane:dichloromethane): 100:0, 100:0, 50:1, 20:1, 10:1, 5:1, 3:1, 1:1, 0:100, 0:100, 0:100, 0:100. The novel compounds were found exclusively in the first 100% dichloromethane fraction (fraction 9), where the main compound had a concentration of 89%. NMR spectra were recorded on a Varian INOVA 500 MHz instrument (Varian, Palo Alto, CA, USA) equipped with a 3 mm ID-PFG-probe. The ^1^H and ^13^C chemical shifts were referenced to solvent signal at δ_H/C_ 7.27/77.0 ppm (CDCl_3_) relative to TMS. All 1D (^1^H, ^13^C) and 2D NMR (gDQFCOSY, ROESY, TOCSY, HOM2DJ, g = gradient enhanced) measurements were performed using standard Varian pulse sequences. Adiabatic broadband and band-selective 2D gradient enhanced HSQC (gHSQCAD) and HMBC (gHMBCAD and bsgHMBC) measurements were performed using standard CHEMPACK 4.0 pulse sequences implemented by K. Krishnamurthy in Varian VnmrJ2.1B software.

The number and nature of unsaturations in the novel compounds were investigated by standard hydrogenation procedures of crude *Cr. modiglianii* extracts using hydrogen and palladium on carbon and rhodium on carbon as catalysts in methanol. In addition, the possible occurrence of triple bonds was checked by hydrogenation using Lindlar’s catalyst in methanol. To determine the presence of primary and/or secondary alcohols, extracts were treated with MSTFA to obtain trimethylsilyl derivatives or with acetic anhydride/pyridine to obtain the corresponding acetates by standard micro derivatization procedures.

### Behavioural experiments

In order to disentangle different sources of recognition cues, we confronted the ants with four different types of cues (henceforth, ‘treatments’): freshly killed workers, their cuticular extracts, a non-polar (containing the hydrocarbons) and a polar fraction (containing the novel compounds) of cuticular extracts. Extracts and fractions were presented on dead, thoroughly solvent-washed ants (‘dummies’). In each test series, we measured whether the observed ants distinguished between intra- and allocolonial cues of the respective partner species (i.e. from the same or a different parabiotic nest). The experiments were performed using a *Cr. modiglianii* colony and *Ca. rufifemur* cues, and vice versa. The potential aggression-reducing effect of the crematoenones was additionally tested using a *Ca. rufifemur* colony and extracts of *Crematogaster difformis* and *Crematogaster coriaria*.

Surface extracts for the behavioural assays were obtained by immersing 50 freeze-killed ants in hexane for ten minutes. Non-polar and polar fractions of these extracts were eluted with distilled hexane, followed by chloroform, using conditioned SiOH columns (CHROMABOND, 100 mg, Macherey-Nagel, Düren, Germany). GC-MS analyses confirmed that the hexane fractions contained only hydrocarbons while the chloroform fractions contained solely the novel compounds. The chloroform of the polar fraction was evaporated, and the fractions were reconstituted in hexane. As dummies we used intracolonial bodies of *Cr. modiglianii* (for *Ca. rufifemur* assays) or *Ca. rufifemur* (for *Cr. modiglianii* assays) that had been extracted with 4 ml hexane, 4 ml chloroform, 4 ml chloroform, and 4 ml hexane for ten min each. Each dummy was treated with an extract quantity equivalent to five individuals. This quantity was chosen to account for potential substance losses during extraction and fractionation; the amount had successfully elicited differential behavioural reactions in earlier experiments [13]. Note that, in the crematoenone addition experiments, the same absolute quantities of hydrocarbons were transferred onto a dummy once with and once without crematoenones.

For the *Ca. rufifemur* assays, a black *Ca. rufifemur* colony (B1) was confronted with treatments from intracolonial *Cr. modiglianii* (B1) and an allocolonial *Cr. modiglianii* (R1, living with a red *Ca. rufifemur*). Secondly, a black *Ca. rufifemur* worker colony (B3) was confronted with intracolonial *Cr. modiglianii* (B3) and allocolonial *Cr. modiglianii* (R3, living with a red *Ca. rufifemur*) treatment series. Finally, a red *Ca. rufifemur* colony (R1) was confronted with *Cr. modiglianii* treatments from the same (R1) and three other colonies. (R3, B1, B3). For the *Cr. modiglianii* assays, we confronted a *Cr. modiglianii* colony (R0) with intracolonial (red *Ca. rufifemur* R0) and two allocolonial treatments (a black and a red *Ca. rufifemur* colony, i.e. B1 and R1).

In the *Ca. rufifemur* assays, the *Cr. modiglianii* dummies were small compared to *Ca. rufifemur* workers. Therefore, we successively held them in front of ten single workers that were walking around in the nest, and categorized each individual reactions as peaceful (antennate), weakly (open mandibles) or strongly aggressive (bite for < 1 s, or bite and keep mandibles locked for > 1 s). In the *Cr. modiglianii* assays, the *Ca. rufifemur* dummy was held with forceps onto the nest trunk in the plastic box so that several *Cr. modiglianii* ants (up to nine) could interact with it simultaneously. During three minutes, each observed interaction was then recorded and classified as above. The observer was not ‘blind’ to the respective treatments, but was unaware of the hypotheses being tested. Within these three minutes, continued interactions were recorded again every 10 sec to provide more weight to long-lasting interactions. This method is consistent with an earlier study [9]. All different treatments were tested in haphazard order and on different places of the nest trunk. For each tested colony, dummies with pure hexane were tested as controls. Ten replicates were carried out per treatment.

The aggression-reducing effect of the crematoenones was further tested using a *Ca. rufifemur* colony (B1) and extracts of *Crematogaster coriaria* and *Crematogaster difformis.* For each extract, we compared the aggression towards the extracts with and without addition of crematoenones (from allocolonial *Cr. modiglianii* R1). The *Cr. difformis* extracts were fractionated over SiOH in order to remove metapleural gland products. This was not necessary in *Cr. coriaria* since their surface extracts only contained hydrocarbons (FM unpublished data).

From each bioassay replicate, we calculated the an aggression index as (*s* + 0.5*w)/*i*, where *s* is the number of strongly aggressive interactions (biting, locking mandibles), *w* is the number of weakly aggressive interactions (opening mandibles), and *i* is the total number of interactions (including peaceful antennation) [41]. The aggression index ranges from 0 (no aggression) to 1 (maximal aggression). We then performed pairwise comparisons between each nestmate and non-nestmate treatment for each test series using generalized linear models (GLMs) with quasibinomial error distribution. The effect of novel compound addition was separately examined using a GLM with quasibinomial error distribution and the explanatory variables ‘extract species’ and ‘crematoenone addition’. The effect size was determined by likelihood ratio tests (F tests). All computations were performed in R Version 2.12.1 [42].

## Competing interests

All authors declare that they have no competing interests.

## Authors’ contributions

Designed research: NB, TS, FM; performed behavioural experiments: FM; performed NMR analysis: TT, JC, UB; performed EI-MS analysis: TS, FM; performed CI-MS, HRMS and reconstructed fragmentation pattern: TB; performed derivatizations: TB, TS; analyzed behavioural data: FM; wrote the paper: FM, TS. All authors read and approved the final manuscript.

## Supplementary Material

Additional file 1: Table S1 List of crematoenones with retention indices and their relative abundance (as percentage of total crematoenones) in the seven studied *Cr. modiglianii* colonies. Percentages >10% are given in bold. The compounds 6, 10 and 18 were further characterized. **Table S2.** GLM results for the influence of crematoenone addition on total aggression of *Ca. rufifemur* B1. Click here for file

Additional file 2 **Chemical analysis of the different crematoenones. Figure S1.** Gas chromatograms of the novel compounds in two different *Cr. modiglianii* colonies. a) colony B3, b) colony B4. Arrows indicate the three compounds further analyzed in Additional file 2: Figure S2. **Figure S2.** HRMS mass spectra of three novel compounds that were further characterized. The spectra were acquired with a JMS-T100GC (GCAccuTOF, JEOL, Japan) time of flight MS. a) compound 6, b) compound 10, c) compound 18 (see Additional file 1: Table S1). Fragments and ions which were tentatively structurally assigned are marked in blue. Tentatively assigned structures (6 and 18) are marked with an asterisk. **Figure S3.** Proposed EI-MS fragmentation of compound 10 (crematoenone). **Figure S4.** Proposed EI-MS fragmentation of compound 6 (dihydrocrematoenone). **Figure S5.** Proposed EI-MS fragmentation of compound 18 (O-acetylcrematoenone). **Figure S6.** GCMS Mass spectra of all 24 crematoenones. The spectra were acquired with a Hewlett Packard 5973 Mass Selective Detector. Click here for file

Additional file 3 **Structure elucidation of the crematoenones. Table S3.** List of molecular masses, accurate masses, empirical formulae and RDBE (ring double bond equivalents) / double bonds. **Table S4.** Results of the NMR analysis of compound 10. Click here for file
