# Supplementary material for: Crematoenones – a novel substance class exhibited by ants functions as appeasement signal
Source: Front Zool. 2013 Jun 6;10:32. doi: 10.1186/1742-9994-10-32 (PMC3691653; doi:10.1186/1742-9994-10-32)
Supplement: Additional file 1: Table S1 — List of crematoenones with retention indices and their relative abundance (as percentage of total crematoenones) in the seven studied Cr. modiglianii colonies. Percentages >10% are given in bold. The compounds 6, 10 and 18 were further characterized. Table S2. GLM results for the influence of crematoenone addition on total aggression of Ca. rufifemur B1. [file 1742-9994-10-32-S1.pdf]

Additional file 1: Supplemental tables

Table S1. List of crematoenones with retention indices and their relative abundance (as percentage of total crematoenones) in the seven studied *Cr. modiglianii* colonies. Percentages >10% are given in bold. The compounds 6, 10 and 18 were further characterized.

peak no.	base peak	retention index	relative abundance in *Cr. modiglianii* cuticular extracts (%)						
			R0	R1	R2	R3	B1	B2	B3
1	43	2038	0.32	0.14	0.42	0.29	0.61	0.45	0.34
2	81	2113	0.11	2.92	**14.84**	0.11	7.10	0.04	0.46
3	69	2132	0.20	0.00	0.00	0.21	0.17	0.18	0.20
4	231	2168	0.21	0.00	0.00	1.22	0.22	0.71	1.30
5	97	2173	0.04	0.61	2.10	0.00	0.29	0.00	0.00
6	71	2180	2.09	0.24	0.30	5.04	1.16	4.55	3.96
7	69	2192	1.40	0.27	0.22	8.89	3.38	3.85	6.69
8	69	2199	0.07	0.00	0.00	0.78	0.18	0.14	0.53
9	231	2202	0.00	0.00	0.00	0.48	0.12	0.02	0.03
10	69	2224	**83.49**	6.19	2.98	**60.73**	**44.90**	**81.20**	**73.20**
11	69	2233	9.57	0.28	0.29	6.04	1.94	6.79	6.75
12	69	2239	0.38	0.35	0.01	5.67	2.47	1.08	0.17
13	91	2365	0.00	0.57	1.36	0.00	0.03	0.00	0.00
14	231	2375	0.17	1.34	**34.70**	0.25	0.38	0.37	0.39
15	231	2383	0.00	0.18	0.72	0.00	0.00	0.00	0.00
16	135	2391	0.00	0.17	0.44	0.00	0.00	0.00	0.00
17	69	2436	0.00	0.00	0.00	0.10	0.00	0.12	0.03
18	69	2447	1.52	0.98	0.41	9.78	**25.40**	0.15	5.62
19	69	2457	0.44	0.00	0.00	0.24	1.55	0.01	0.05
20	231	2516	0.00	2.19	2.59	0.00	0.00	0.00	0.00
21	285	2526	0.00	2.17	9.40	0.00	9.67	0.00	0.00
22	87	2558	0.00	4.54	4.55	0.04	0.06	0.01	0.05
23	231	2571	0.00	7.62	3.92	0.00	0.11	0.01	0.04
24	85	2577	0.00	**69.25**	**20.74**	0.13	0.27	0.25	0.18

Table S2. GLM results for the influence of crematoenone addition on total aggression of *Ca. rufifemur* B1.

Parameter	Deviance	*df*	*F*	*P*
crematoenone addition	57.3	1	20.87	< 0.0001
species	5.4	2	0.98	0.38
species : crematoenone addition	23.3	2	4.71	0.013
residual error	155.9	56		
total	242.0	61		
