# Supplementary material for: Crematoenones – a novel substance class exhibited by ants functions as appeasement signal
Source: Front Zool. 2013 Jun 6;10:32. doi: 10.1186/1742-9994-10-32 (PMC3691653; doi:10.1186/1742-9994-10-32)
Supplement: Additional file 2 — Chemical analysis of the different crematoenones. Figure S1. Gas chromatograms of the novel compounds in two different Cr. modiglianii colonies. a) colony B3, b) colony B4. Arrows indicate the three compounds further analyzed in Additional file 2: Figure S2. Figure S2. HRMS mass spectra of three novel compounds that were further characterized. The spectra were acquired with a JMS-T100GC (GCAccuTOF, JEOL, Japan) time of flight MS. a) compound 6, b) compound 10, c) compound 18 (see Additional file 1: Table S1). Fragments and ions which were tentatively structurally assigned are marked in blue. Tentatively assigned structures (6 and 18) are marked with an asterisk. Figure S3. Proposed EI-MS fragmentation of compound 10 (crematoenone). Figure S4. Proposed EI-MS fragmentation of compound 6 (dihydrocrematoenone). Figure S5. Proposed EI-MS fragmentation of compound 18 (O-acetylcrematoenone). Figure S6. GCMS Mass spectra of all 24 crematoenones. The spectra were acquired with a Hewlett Packard 5973 Mass Selective Detector. [file 1742-9994-10-32-S2.pdf]

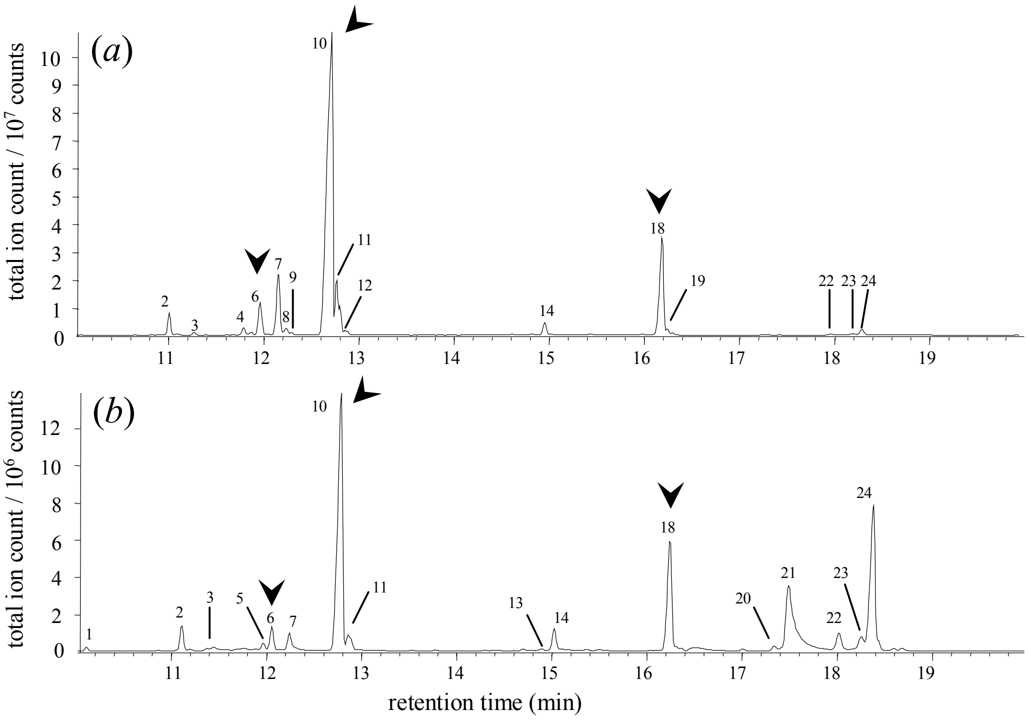

Fig. S1 Gas chromatograms of the novel compounds in two different *Cr. modiglianii* colonies. a) colony B3, b) colony B4. Arrows indicate the three compounds further analyzed in Fig. S2.

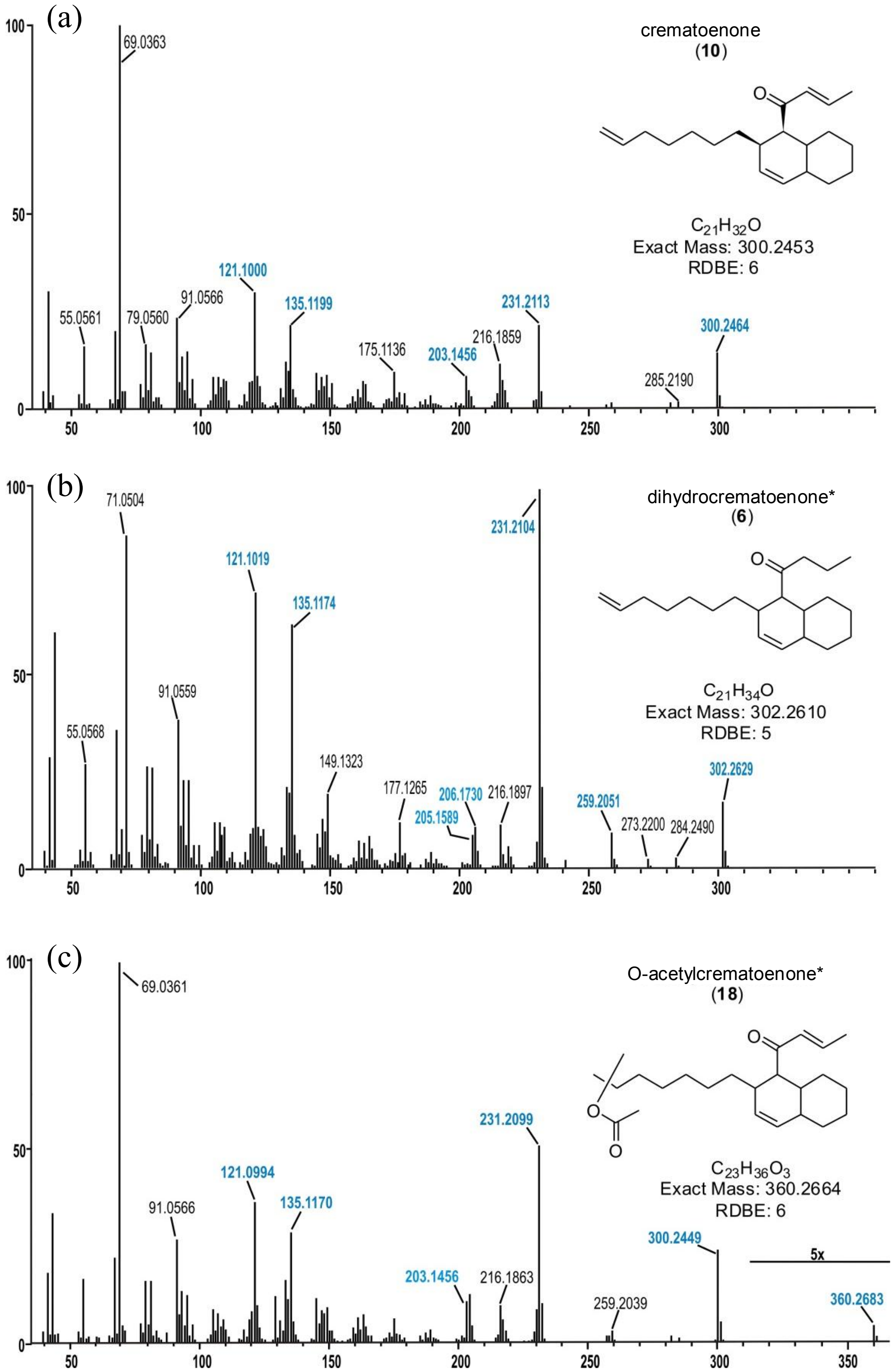

Fig. S2 HRMS Mass spectra of three novel compounds that were further characterized. a) compound **6**, b) compound **10**, c) compound **18**. Fragments and ions which were tentatively structurally assigned are marked in blue. Tentatively assigned structures (**6** and **18**) are marked with an asterisk.

Fig. S3 Proposed EI-MS fragmentation of compound **10** (crematoenone).

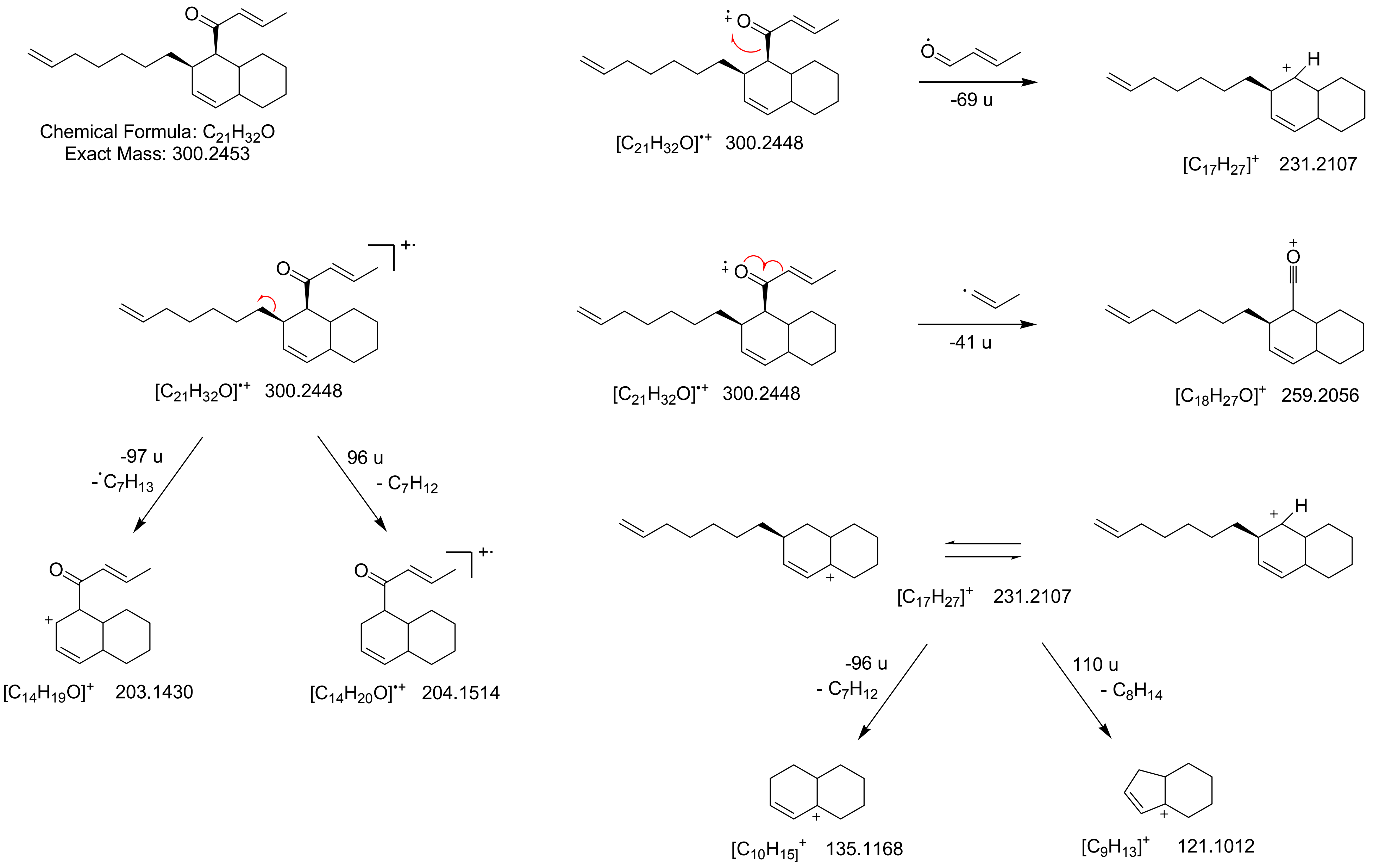

Fig. S4 Proposed EI-MS fragmentation of compound **6** (dihydrocrematoenone).

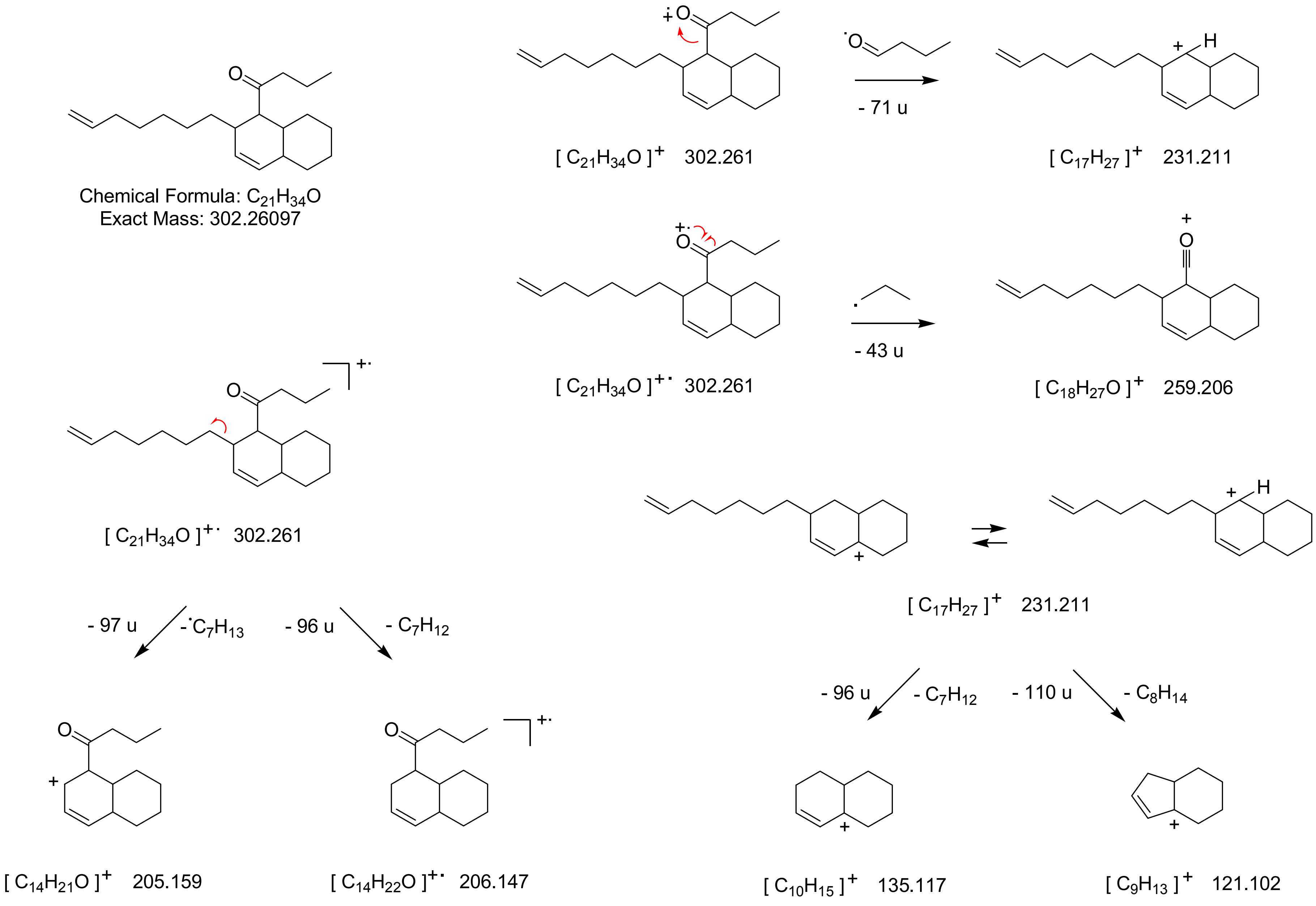

Fig. S5 Proposed EI-MS fragmentation of compound **18** (O-acetylcrematoenone).

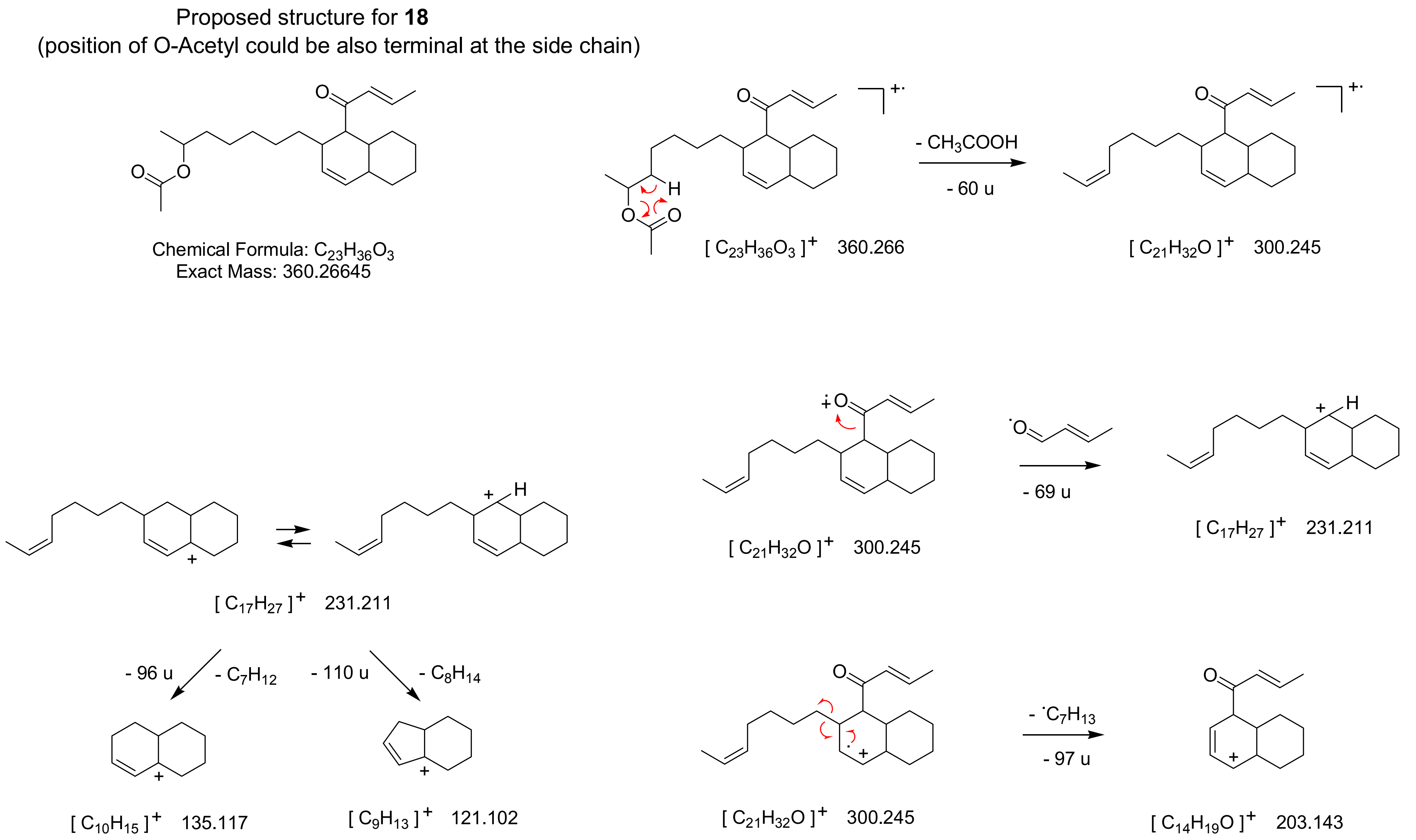

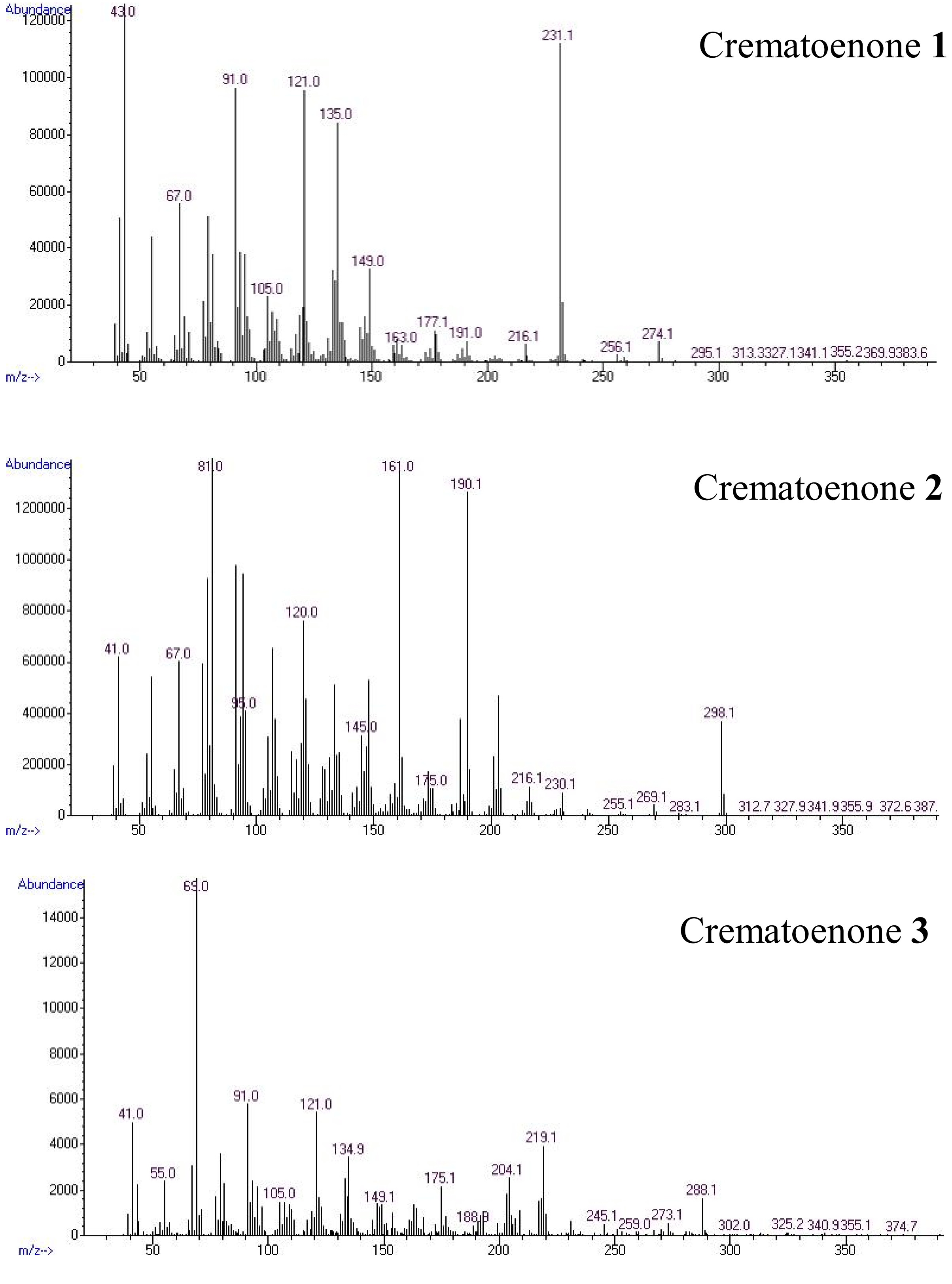

Fig. S6a. GCMS mass spectra of Crematoenones 1 to 3.

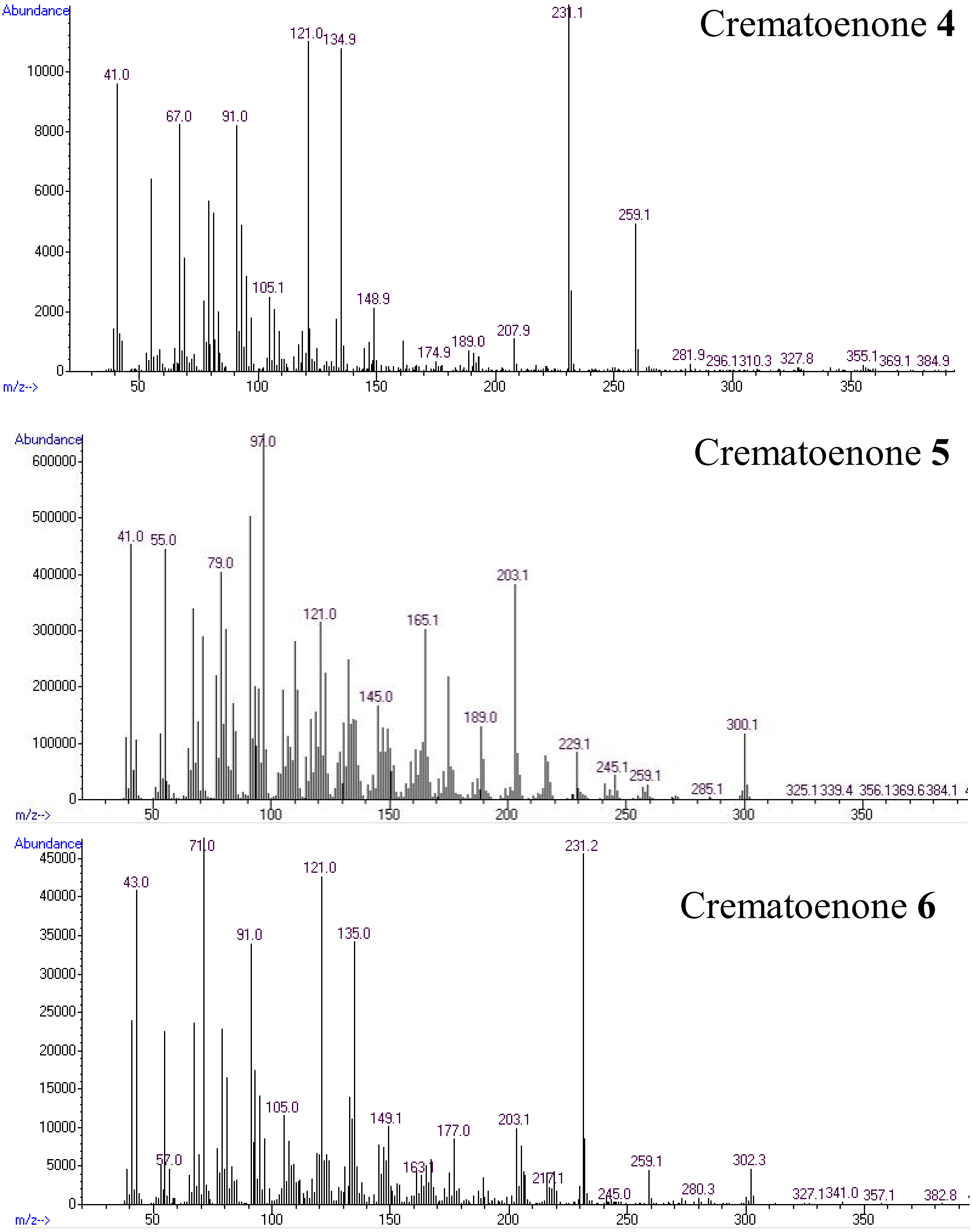

Fig. S6b. GCMS mass spectra of Crematoenones 4 to 6.

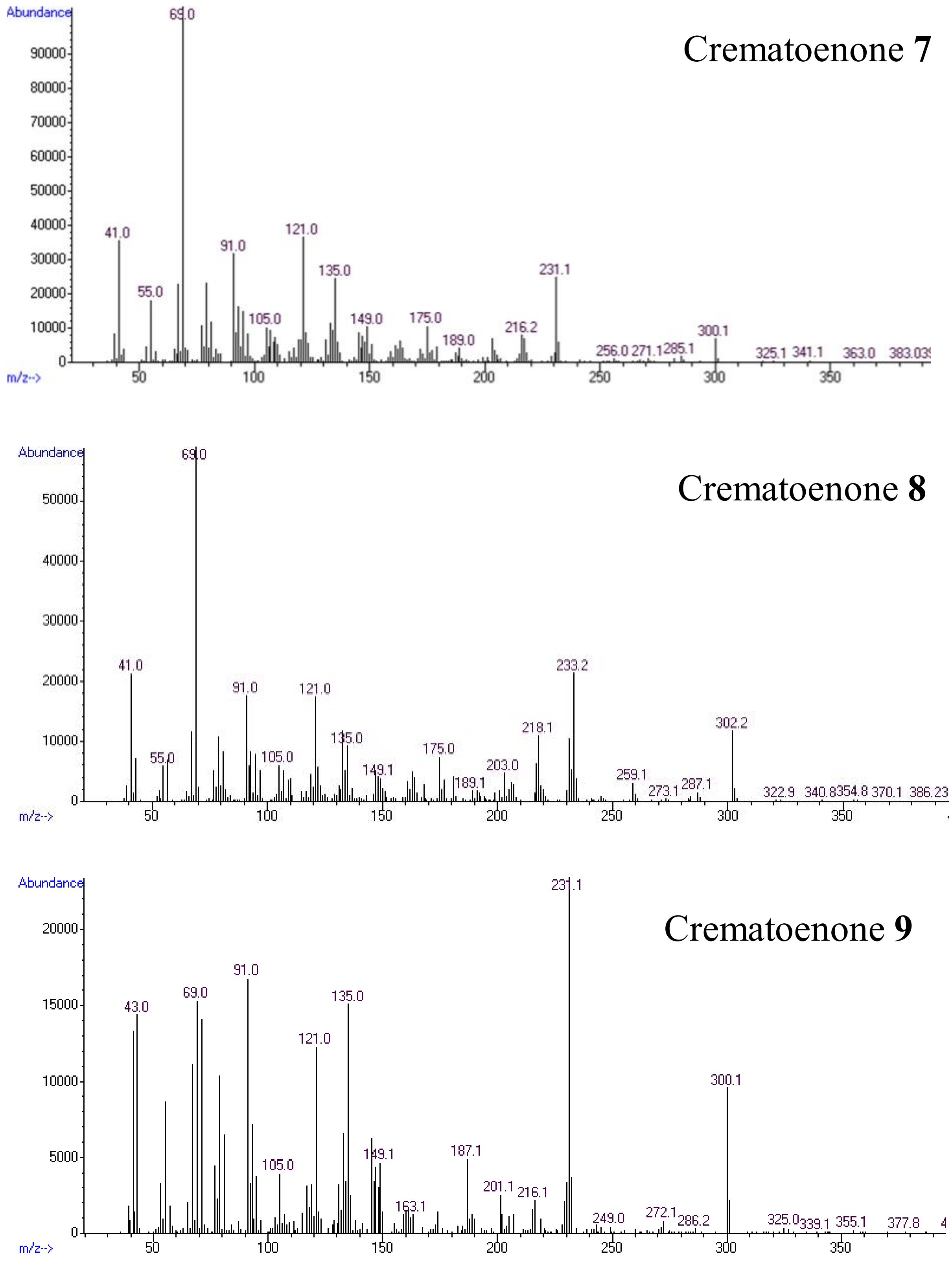

Fig. S6c. GCMS mass spectra of Crematoenones 7 to 9.

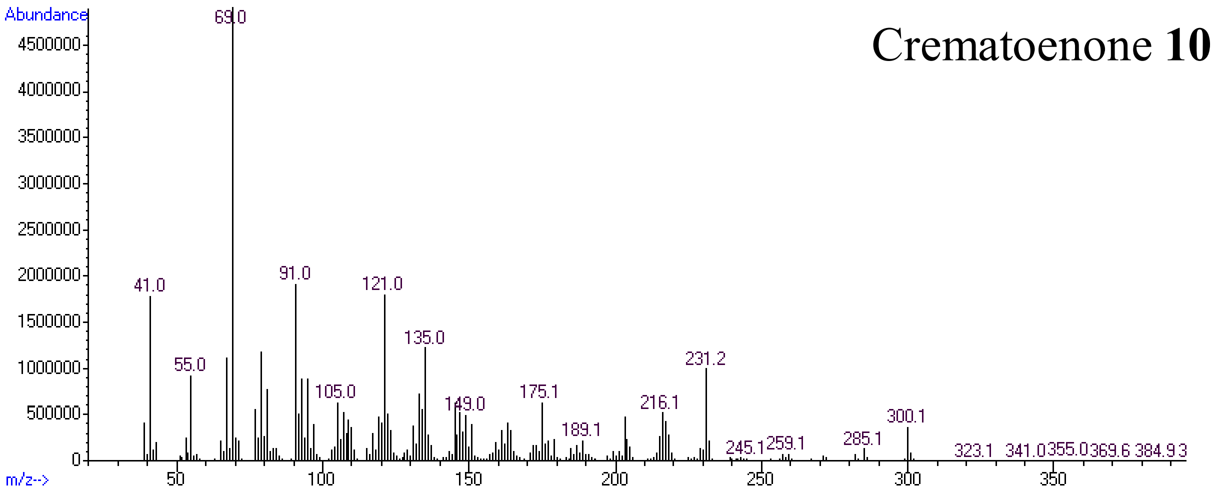

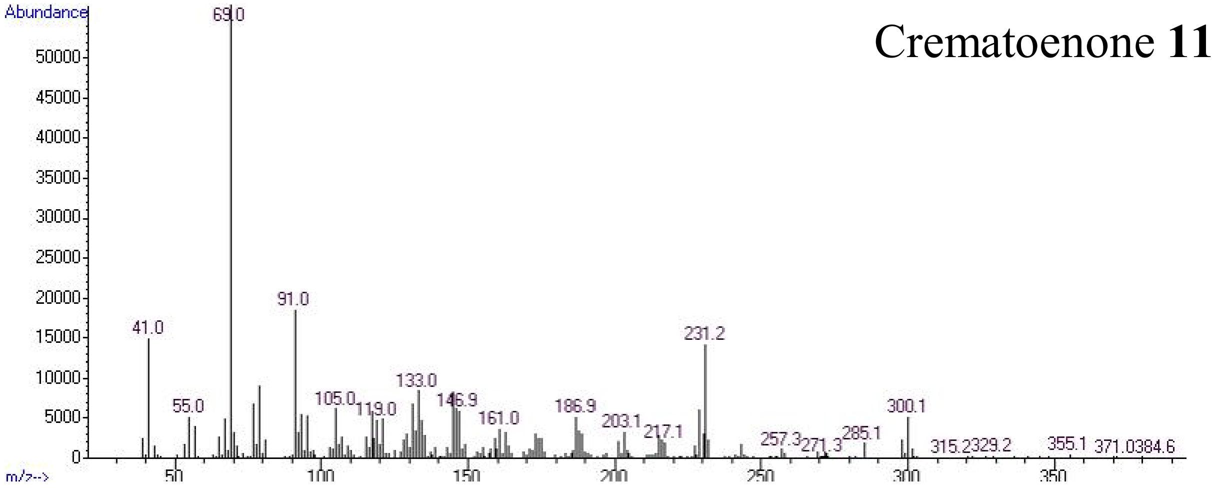

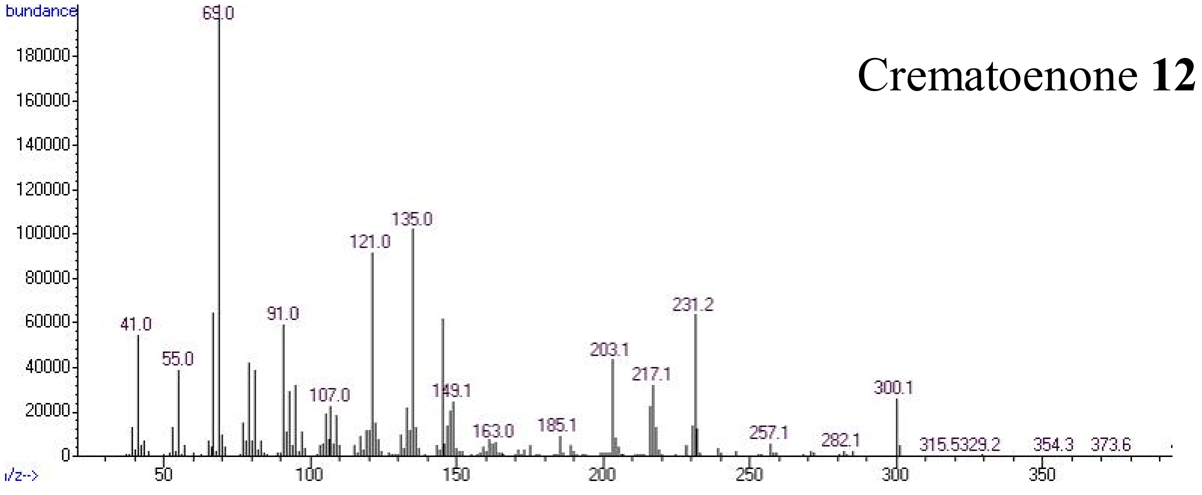

Fig. S6d. GCMS mass spectra of Crematoenones 10 to 12.

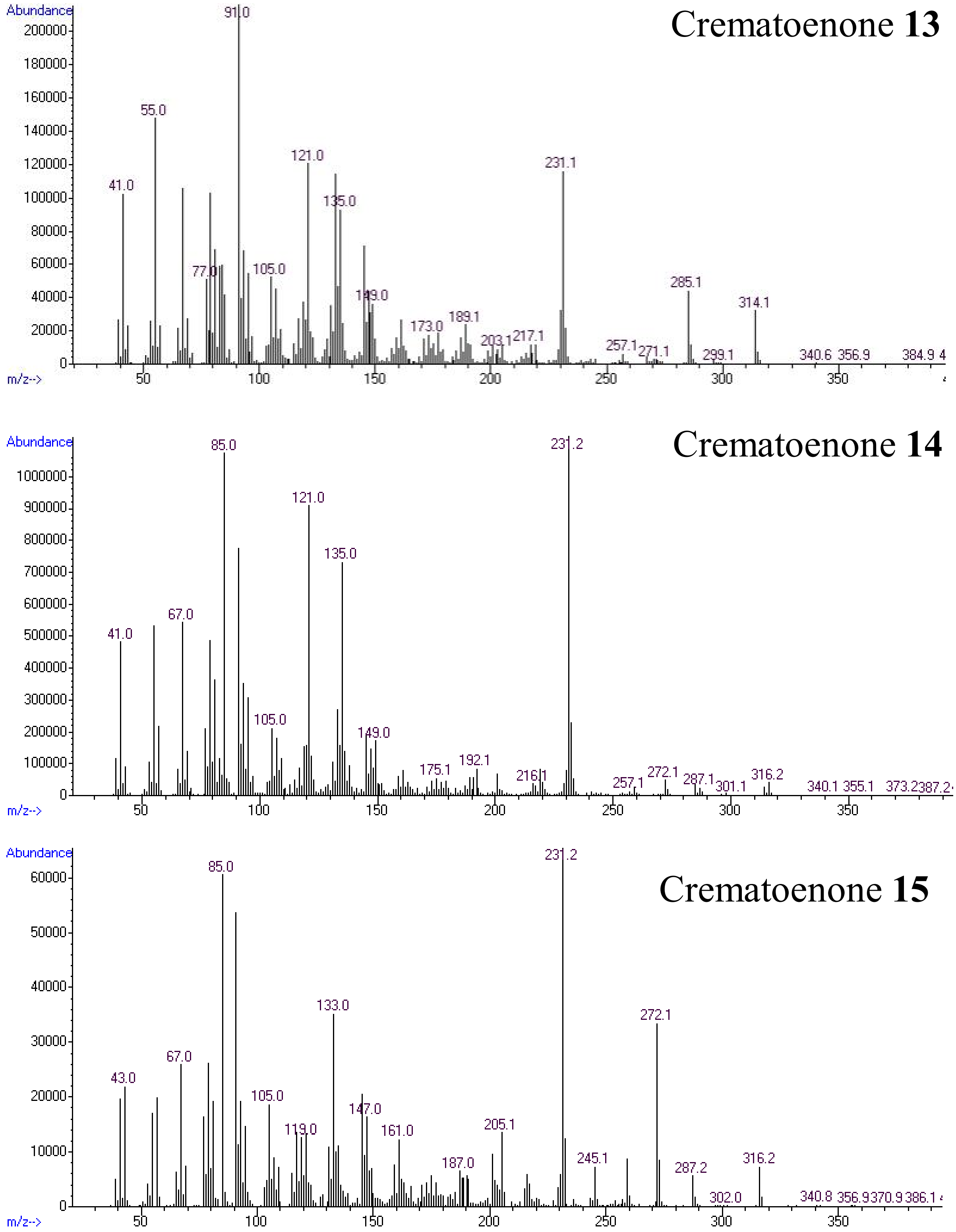

Fig. S6e. GCMS mass spectra of Crematoenones 13 to 15.

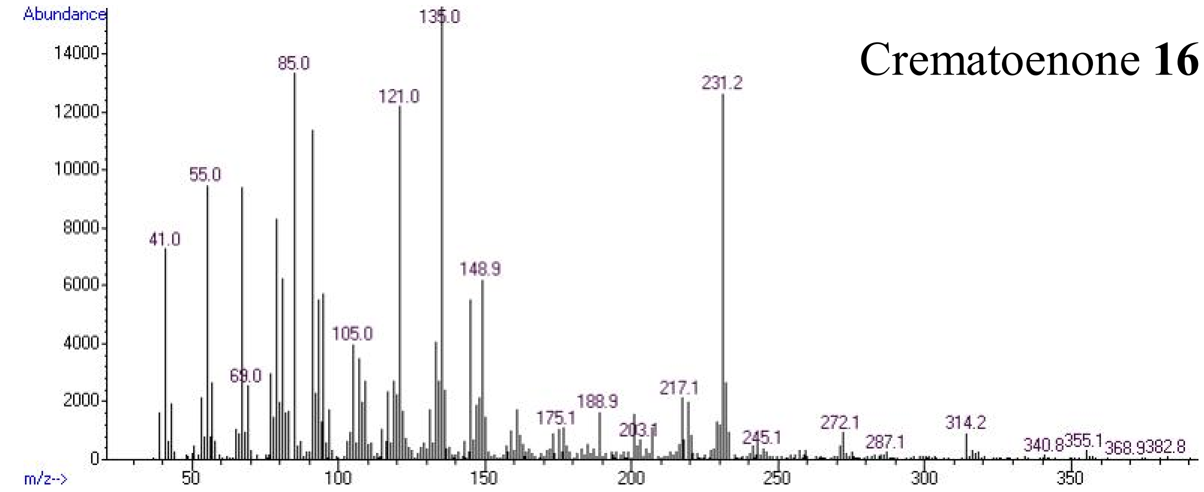

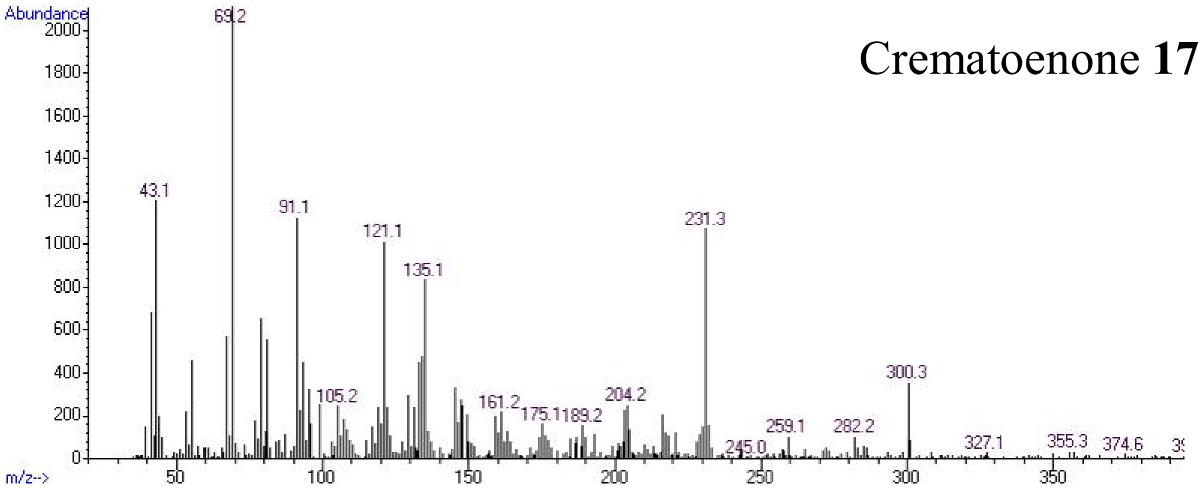

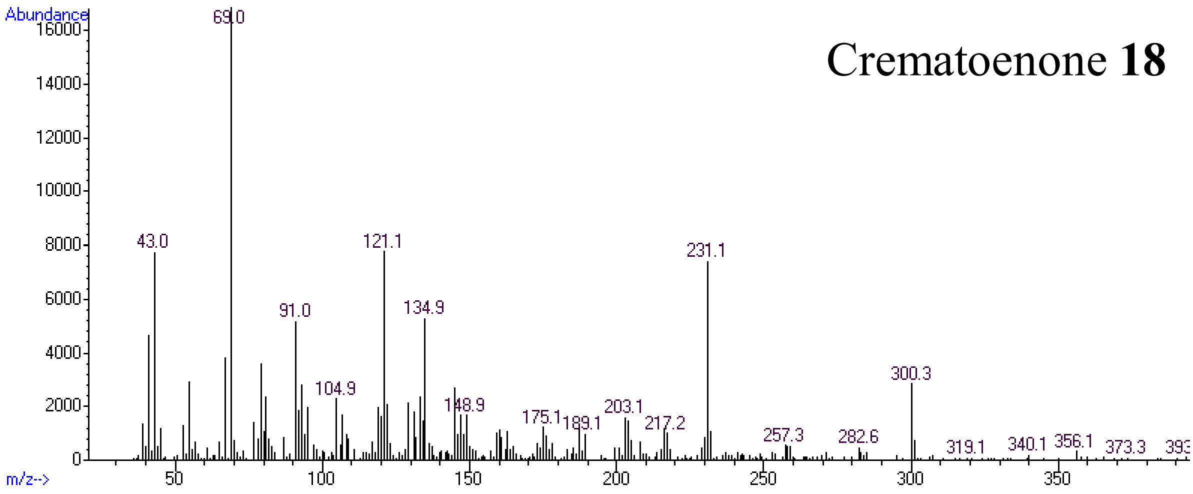

Fig. S6f. GCMS mass spectra of Crematoenones 16 to 18.

Crematoenone **19**

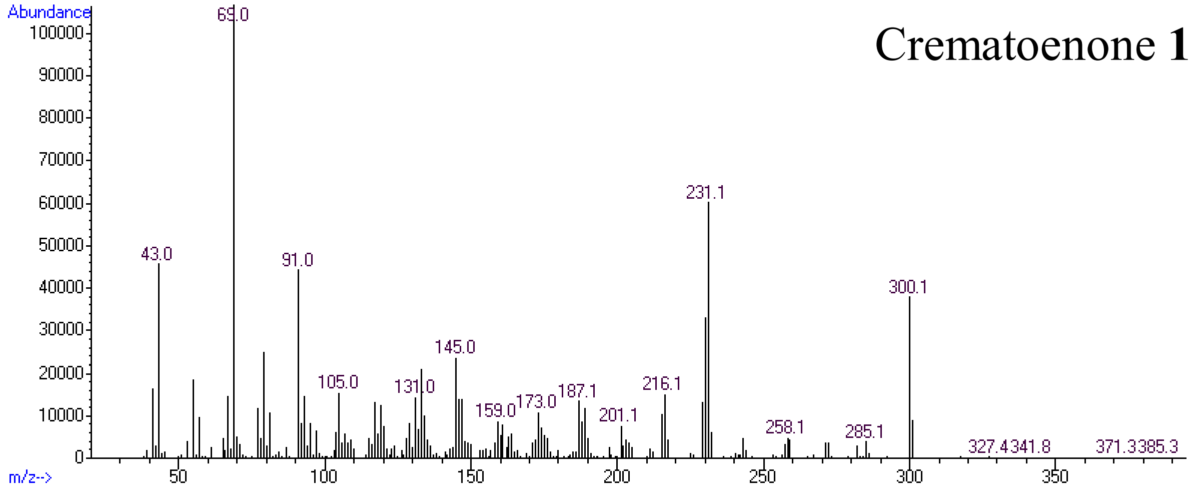

Crematoenone **20**

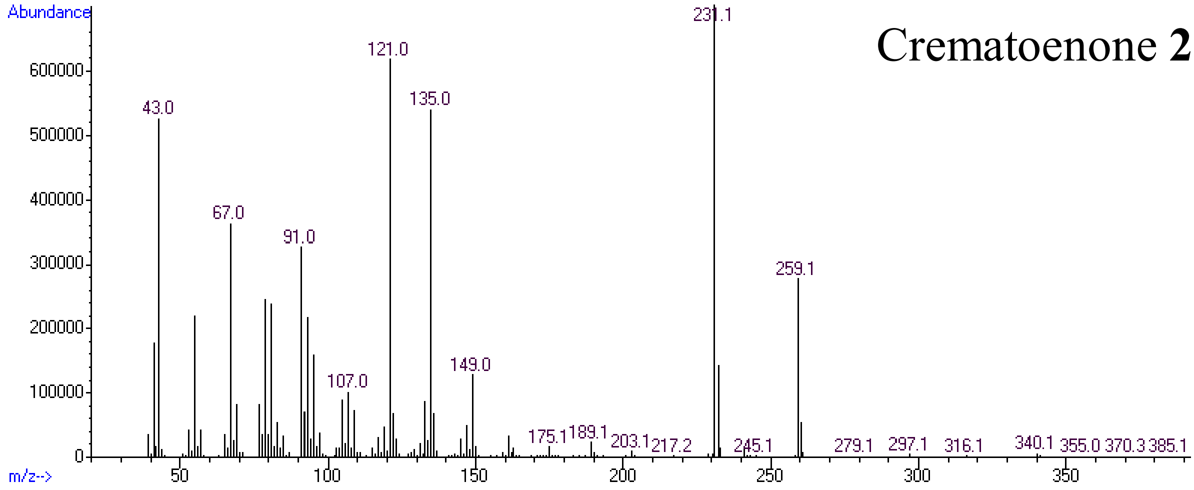

Crematoenone **21**

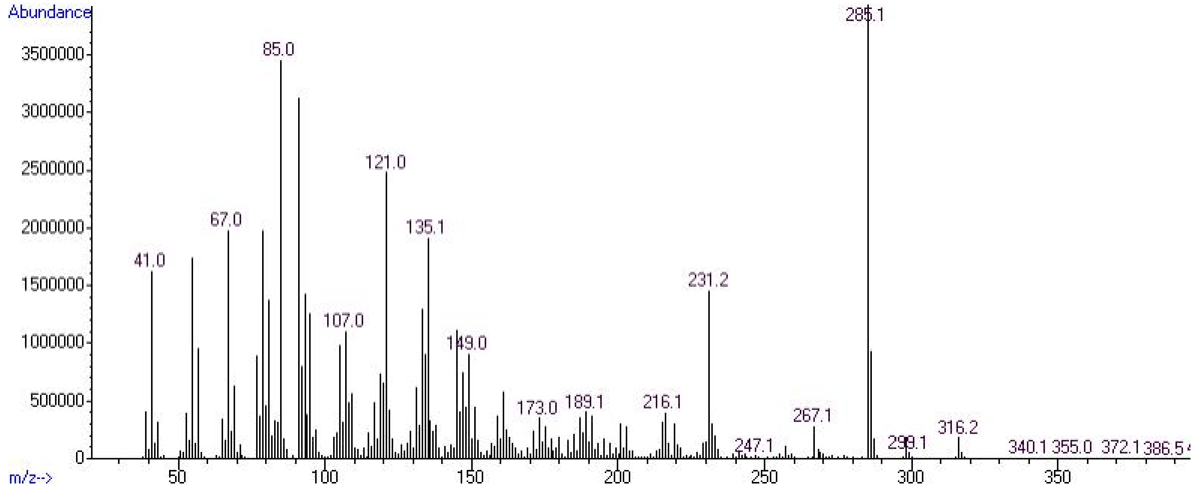

Fig. S6g. GCMS mass spectra of Crematoenones 19 to 21.

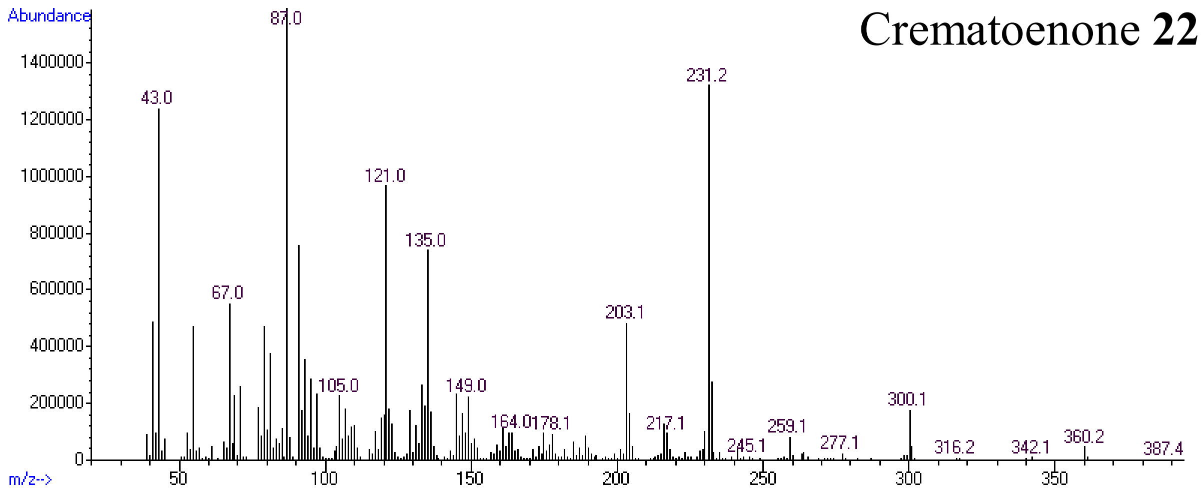

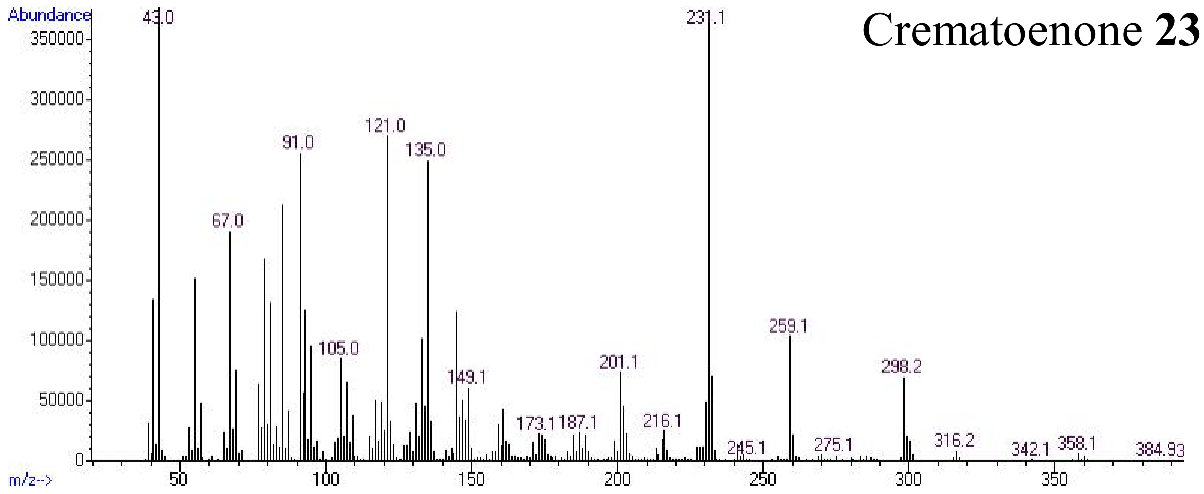

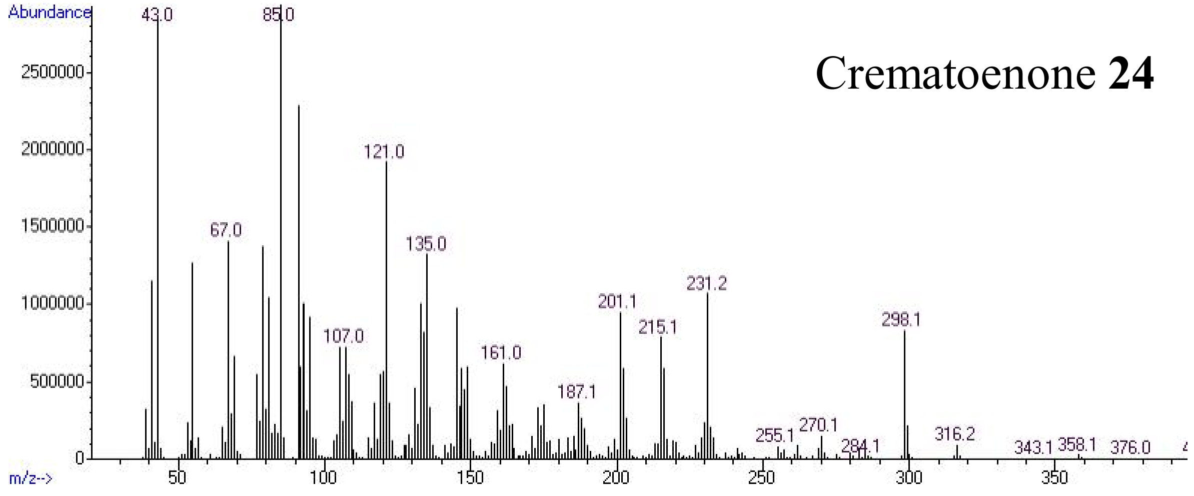

Fig. S6h. GCMS mass spectra of Crematoenones 22 to 24.
