# Supplementary material for: Crematoenones – a novel substance class exhibited by ants functions as appeasement signal
Source: Front Zool. 2013 Jun 6;10:32. doi: 10.1186/1742-9994-10-32 (PMC3691653; doi:10.1186/1742-9994-10-32)
Supplement: Additional file 3 — Structure elucidation of the crematoenones. Table S3. List of molecular masses, accurate masses, empirical formulae and RDBE (ring double bond equivalents) / double bonds. Table S4. Results of the NMR analysis of compound 10. [file 1742-9994-10-32-S3.pdf]

Additional file 3: Structure elucidation of the novel compounds

The three compounds which were further characterized possessed five (compound **6**) or six (compounds **10** and **18**) ring/double bond equivalents (RDBE). Two (compounds **6**, **18**) or three RDBE (compound **10**) could be hydrogenated, leaving three (**6**, **10**) or four (**18**) RDBEs that could not be hydrogenated. As revealed by CI-MS, the m/z values in the high mass range of the EI-MS represented the molecular ion peaks of the corresponding compounds. Hence, their elemental compositions were calculated based on HRMS (Table S3). The data indicate one (compounds **6** and **10**) or three (compound **18**) oxygen atoms in the respective molecules. Treatment with MSTFA or with acetic anhydrid did not result in changes of their retention indices and mass spectra. These failed derivatizations made primary or secondary hydroxyl groups seem improbable.

All compounds were available in limited amounts, and in complex mixtures. Thus, ^{1}H and ^{13}C NMR data and ROESY and HMBC correlations could be obtained only for the major compound **10** (Table S4). The structure elucidation revealed a *trans*-1,2,4a,5,6,7,8,8a-octahydronaphtalene subunit whose ^{1}H and ^{13}C chemical shifts strongly agreed with literature data for a similar substructure (Solanopyrone D: [2], UCS1025A and B: [3]). Carbon atoms 1'-4' represent a 2-butenoyl substituent with an *E*-configurated double bond indicated by $J_{2'\text{-H}, 3'\text{-H}}$ 15.5 Hz, which is connected to C-1 of the octahydronaphtalene as shown by the strong HMBC correlations between H-1 and C-1'. The remaining seven carbon atoms (C-1"- C-7") form an alkenyl moiety with a terminal double bond. HMBC correlations between 2-H and C-1" as well as C-2" unequivocally reveal that the alkenyl substituent is linked to the octahydronaphthalene unit via C-2. The relative configuration was determined by ROESY and was unambiguously assigned as 1R*,2R*,4aS*,8aR* (Table S4; Figure 2).

The EI-HRMS measurements allowed the assignment of the elemental composition of fragment ions and losses. Based on the NMR-assigned structure of compound **10**

(crematoenone) and the HRMS results, fragmentation patterns and cleavages for this compound were established. The proposed key fragments for compounds **6, 10** and **18** are shown in Figures S3-S5. The main diagnostic fragment ions (m/z 121, 135, 203, 204, 231, 300) could be assigned and were verified by the elemental compositions from the HRMS measurements.

The fragmentation mechanisms of **10** were applied for the analysis of the related compounds **6** and **18** and allowed sound proposals for the chemical structures of **6** and **18**. Compared to crematoenone (**10**) the interpretation of the GC-HRMS data showed that compound **6** (Figure S1a) is a hydrogenated derivative of **10** (mass difference +2 u and one RDBE less). The key ions 121, 135, 205, 206, 231, 259, and 302 strongly suggested that the differences were due to the loss of the double bond in the 2-butenoyl substituent (Figures S3-S5). Hence, we tentatively identified compound **6** as 2',3'-dihydrocrematoenone. For compound **18**, HRMS showed a mass difference of +60 u (reflecting $C_2H_4O_2$) compared to **10** but no change in the RDBE. This can be explained by the replacement of a double bond by an O-Acetyl functionality. The significant similarity to **10** (especially the fragment ions m/z 203, 204) strongly indicated that the modification took place at the alkenyl moiety, while the rest of the molecule remains unaffected. The position of the O-acetyl group cannot be determined by MS, but position 6" or 7" seems likely.

Table S3. List of molecular masses, accurate masses, empirical formulae and RDBE (ring double bond equivalents) / double bonds.

peak no.	retention index	M^+ (EI)	CI-MS molecular mass results	accurate mass	empirical formula	RDBE / double bonds
6	2180	302	302	302.2571	$C_{21}H_{34}O_1$	5 (2 double bonds)
10	2224	300	300	300.2470	$C_{21}H_{32}O_1$	6 (3 double bonds)
18	2447	360	360	360.2713	$C_{23}H_{36}O_3$	6 (2 double bonds)

Table S4 Results of the NMR analysis of compound **10**.

Pos.	δ_C	mult	Proton	δ_H	J (Hz)	HMBC[a]	ROESY[b]
1	53.60	CH	1-H	2.94	dd, J = 5.9, 11.3	C-2, 4a, 8, 8a, 1', 1"	2-H, 4a-H, 8-H_{ax}
2	37.02	CH	2-H	2.33	m	C-3, 1", 2"	1-H, 3-H
3	128.73	CH	3-H	5.71	ddd, J = 2.5, 4.7, 10.0	C-1, 2, 4a	2-H, 2''-H
4	131.89	CH	4-H	5.45	d, J = 10.0	C-2, 4a, 8a, 5	5-H_{eq}
4a	42.73	CH	4a-H	1.71	br t, J ≈ 12 [c]		1-H, 8-H_{ax}
5	33.07	CH_2	H_{eq}	1.74 ov	br d, J ≈ 13,4 [c]		4-H
			H_{ax}	1.10	dddd, J = 3.4,12.3,12.3,12.3		
6	26.82	CH_2	H_{eq}	1.75	br d-like, J ≈ 13 [c]		
			H_{ax}	1.30 ov	q-like, J ≈ 13.9 [c]		
7	26.53	CH_2	H_{eq}	~1.72 ov	br d-like, J ≈ 13 [c]		
			H_{ax}	1.36 ov	q-like, J ≈ 13,8 [c]		8a-H
8	29.94	CH_2	H_{eq}	1.92	br d, J = 13.4 [c,d]		8a-H
			H_{ax}	0.72	dddd, J = 3.5, 12.0, 12.0, 12.0 [c,d]	C-1, 4a, 7, 8a	1-H, 4a-H
8a	36.99	CH	8a-H	1.53	dddd, J= 3.1, 11.0, 11.0, 11.0 [c]		1"-H, 7-H_{ax}, 8-H_{eq}
1'	201.49	C					
2'	132.56	CH	2'-H	6.13	dq, J = 1.7, 15.7	C-1, 1', 4'	4'-H
3'	141.83	CH	3'-H	6.85	dq, J = 6.6, 15.3	C-1', 2', 4'	
4'	18.19	CH_3	4'-H_3	1.89	dd, J = 1.7, 6.8 [c]	C-1', 2', 3'	2'-H
1"	32.19	CH_2	1"-H_2	1.19 ov	m		8a-H
2"	27.14	CH_2	2"-H_2	1.18 ov	m		2-H
3"	29.26	CH_2	3"-H_2	1.25	m		
4"	28.79	CH_2	4"-H_2	1.34 ov	m		
5"	33.73	CH_2	5"-H_2	2.00	bq-like, J ≈ 6.8	C-4", 3", 6", 7"	
6"	139.09	CH	6"-H	5.78	ddt, J = 6.5,10.2,17.9	C-4", 5"	
7"	114.10	CH_2	7"-Ha	4.91	br d, J = 10.4	C-5"	
			7"-Hb	4.97	dq, J = 1.9,17.2	C-5"	

[a] Observed ^1H-^{13}C long-range correlations in HMBC spectra (500 MHz, $CDCl_3$)

[b] Selected ROESY correlations (500 MHz, $CDCl_3$)

[c] Coupling constants extracted from 1D traces of the coupled bsgHSQCAD spectrum

[d] Coupling constants from Homo 2DJ spectrum

J: Observed coupling constants were not averaged; ov: fully or partially overlapped by other signals

References

1. Menzel F, Blüthgen N, Schmitt T: **Tropical parabiotic ants: Highly unusual cuticular substances and low interspecific discrimination.** *Frontiers in Zoology* 2008, **5:**16.
2. Oikawa H, Yokota T, Sakano C, Suzuki Y, Naya A, Ichihara A: **Solanopyrones, Phytotoxins produced by *Alternaria solani*: Biosynthesis and isolation of minor components.** *Bioscience, Biotechnology and Biochemistry* 1998, **62:**2016-2022.
3. Agatsuma T, Akama T, Nara S, Matsumiya S, Nakai R, Ogawa H, Otaki S, Ikeda S, Saitoh Y, Kanda Y: **UCS1025A and B, new antitumor antibiotics from the fungus *Acremonium* Species.** *Organic* 2002, **4:**4387-4390.
